# Naturally Occurring Cinnamic Acid Sugar Ester Derivatives

**DOI:** 10.3390/molecules21101402

**Published:** 2016-10-24

**Authors:** Yuxin Tian, Weirui Liu, Yi Lu, Yan Wang, Xiaoyi Chen, Shaojuan Bai, Yicheng Zhao, Ting He, Fengxue Lao, Yinghui Shang, Yu Guo, Gaimei She

**Affiliations:** 1 School of Chinese Pharmacy, Beijing University of Chinese Medicine, Beijing 100102, China; tianyuxin1216@163.com (Y.T.); liuweirui2012@126.com (W.L.); 18739908461@163.com (Y.W.); chenxiaofly1209@126.com (X.C.); shaojuanbai@163.com (S.B.); zhaoyicheng0824@163.com (Y.Z.); 13580771726@163.com (T.H.); 2 School of Basic Medicine, Beijing University of Chinese Medicine, Beijing 100102, China; luyi780707@hotmail.com; 3 Beijing Key Laboratory of Bioactive Substances and Functional Foods, Beijing Union University, Beijing 100191, China; Fengxue@buu.edu.cn (F.L.); Yinghui@buu.edu.cn (Y.S.); guoyu@buu.edu.cn (Y.G.)

**Keywords:** cinnamic acid sugar ester derivatives, phytochemistry, pharmacological activity, traditional Chinese medicine

## Abstract

Cinnamic acid sugar ester derivatives (CASEDs) are a class of natural product with one or several phenylacrylic moieties linked with the non-anomeric carbon of a glycosyl skeleton part through ester bonds. Their notable anti-depressant and brains protective activities have made them a topic of great interest over the past several decades. In particular the compound 3′,6-disinapoylsucrose, the index component of Yuanzhi (a well-known Traditional Chinese Medicine or TCM), presents antidepressant effects at a molecular level, and has become a hotspot of research on new lead drug compounds. Several other similar cinnamic acid sugar ester derivatives are reported in traditional medicine as compounds to calm the nerves and display anti-depression and neuroprotective activity. Interestingly, more than one third of CASEDs are distributed in the family *Polygalaceae*. This overview discusses the isolation of cinnamic acid sugar ester derivatives from plants, together with a systematic discussion of their distribution, chemical structures and properties and pharmacological activities, with the hope of providing references for natural product researchers and draw attention to these interesting compounds.

## 1. Introduction

**Figure 1:**
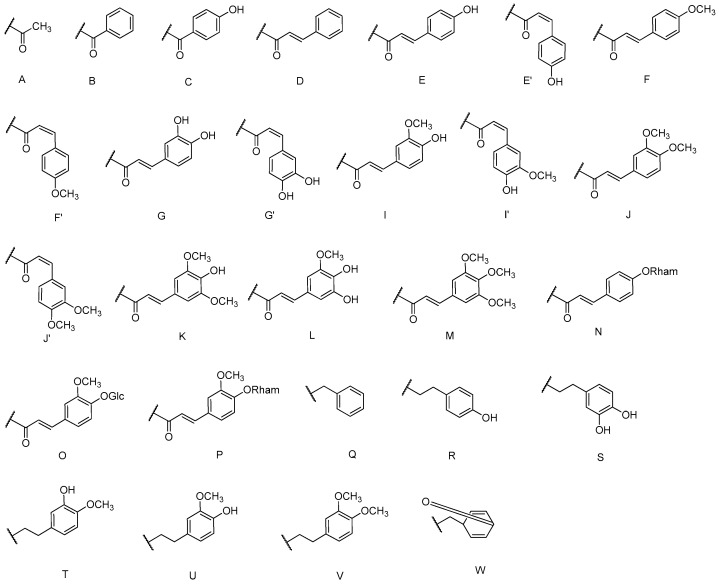
Substituent groups.

As a class of natural products, cinnamic acid sugar ester derivatives (CASEDs) have become a research focus owing to their structural diversity, together with distinctive and remarkable pharmacodynamic actions, such as anti-depression, anti-cancer, anti-oxidant, anti-inflammatory and anti-viral activities [1,2,3,4,5]. They have one or more phenylacrylic (Ph-CH=CH-CO-) moieties or their derivatives linked to the non-anomeric carbon skeletons of the glycosyl part through ester linkage-bonds. The phenylacrylic group, also named cinnamic acid part, may usually contain hydroxyl or methoxy substituted groups (Figure 1). The aglycone group is the core structure, and includes monosaccharides, disaccharides, trisaccharides, tetrasaccharides, pentasaccharides, hexsaccharides and heptasaccharides. There are one or several -OH groups on the non-anomeric carbon skeleton, connected with the cinnamic acid moiety.

Since 1968 [6], more than 330 CASEDs have been found in the medicinal plants of the families *Polygalaceae*, *Scrophulariaceae*, *Liliaceae*, *Oleaceae*, *Bignoniaceae*, *Polygonaceae*, *Orobanchaceae*, *Rosaceae*, *Lamiaceae*, *Labiatae*, *Gesneriaceae*, *Rubiaceae*, *Cruciferae*, *Plantaginaceae*, *Verbenaceae*, *Magnoliaceae*, *Amaranthaceae*, *Smilacaeae*, *Sterculiaceae*, *Hymenophyllaceae* and *Asclepiadaceae* (Table 1). Interestingly, more than one third of CASEDs are distributed in the family *Polygalaceae*, which is used for tranquilizing the mind and promoting intelligence as in Traditional Chinese Medicine (TCM) [1]. Yuanzhi, the dried root of *Polygala tenuifolia*, a representative plant from the *Polygalaceae*, is a well-known TCM used for its sedative, psychotic, cognitive and depressant effects. It is used in the clinic for tranquilizing and reinforcing the mind, and is commonly applied to physical and mental illness.

The oligosaccharide cinnamic acid esters are regarded as the predominant active antidepressant ingredients. 3′,6-Disinapoylsucrose (DISS, **73**), as the index component of Yuanzhi, has been studied to the level of the molecular mechanism of its antidepressant effects, representing a hotspot of research on new drug precursor compounds [7]. There are also other multiple reports [8,9] on the antidepressant effects of sibiricose A5 (**28**) and tenuifoliside A (**51**). There are additionally several active compounds from *Scrophulariae Radix*, *Rehmannia Radix*, *Smilacis China Rhizoma*, which according to common wisdom, calm the nerves with anti-depression and neuroprotective activity (Table 2).

Up to now, there is no relevant literature that analyzes all those CASED compounds systematically. Therefore, this paper is aimed at systematically clarifying the distribution, chemical structures and pharmacological activities of CASEDs, in the hope of drawing more researchers’ attention to these interesting substances.

## 2. Chemical Constituents

Cinnamic acid sugar ester derivatives (CASEDs) are an important type of natural product. Structurally, they have a glycosyl linked with the phenylacrylic group using ester bonds. The glycosyl part maybe contain one, or several sugar units, which are attached via an -OH group to another -OH by condensation reactions. So far, glucopyrannosyl, rhamnopyranosyl, fructofuranosyl, arabinopyranosyl, galactopyranosyl, apiofuranosyl, xylopyranosyl, lyxopyranosyl, allopyranosyl, fucopyranosyl and lactopyranosyl moieties have been reported to occur in CASEDs. The glycosyl portion usually has an anomeric carbon of one sugar connected to the C-2, C-3 and C-4 of the other glycosyl group. Here, the non-anomeric carbon of the glycosyl part connected (Table 3).

Up to now, there has been no detailed research on the extraction procedures for these chemical constituents. Generally, the crude extracts wer prepared with different concentrations of methanol, ethanol or acetone-water solution by the impregnation method, refluxing extraction or decoction method [10,11,12,13,14,15,16,17,18,19,20,21,22,23,24,25,26,27,28,29,30,31,32,33,34,35,36,37,38,39,40,41,42,43,44,45,46,47,48,49,50,51,52,53,54,55,56,57,58,59,60,61,62,63,64,65,66,67,68,69,70,71,72,73,74,75,76,77,78,79,80,81,82,83,84,85,86,87,88,89,90,91,92,93,94,95,96,97,98,99,100,101,102,103,104,105,106,107,108,109,110,111]. Then the extracts were evaporated in a rotary evaporator to yield a syrupy residue. This residue was suspended in H_2_O and extracted successively with petroleum ether, CHCl_3_, EtOAc and H_2_O-satd *n*-BuOH [10,14,15,22]. The different extracts were then fractionated on different chromatographic columns with different mobile phases. Thereinto, silica gel CC was the most commonly used positive phase chromatographic column and eluted with petroleum ether, petroleum ether–EtOAc CHCl_3_–EtOAc, CHCl_3_–MeOH, CHCl_3_–MeOH–H_2_O with various ratios [10,11,22,30]. Mitsubishi Diaion HP-20, Diaion HP20SS, Chromatorex ODS, different types of macroporous resin and MCI columns were the reverse phase chromatography columns, which were used widely, eluted with a step-gradient of MeOH–H_2_O or EtOH–H_2_O (10%–100%), respectively. Sephadex LH-20 was also commonly used [19,31]. Some oligosachariches were isolated by preparative HPLC (Develosil Lep-ODS) [11]. Preparative TLC and recycle semi-preparative HPLC were often used to further purify samples [15].

### 2.1. Monosaccharide Esters

**Figure 2:**
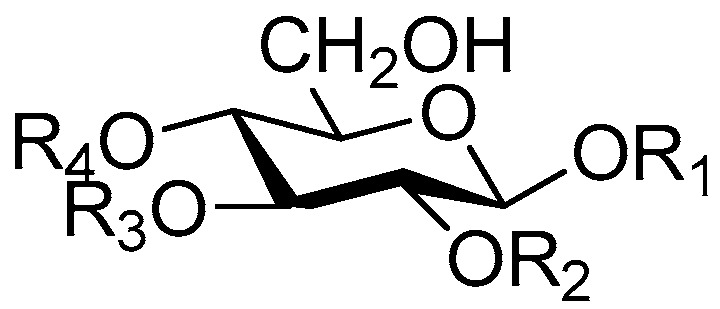
Structures of compounds **1**–**10**.

**Figure 3:**
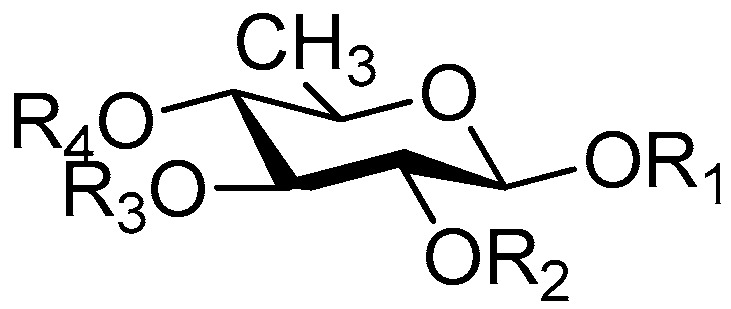
Structures of compounds **11**–**15**.

**Figure 4:**
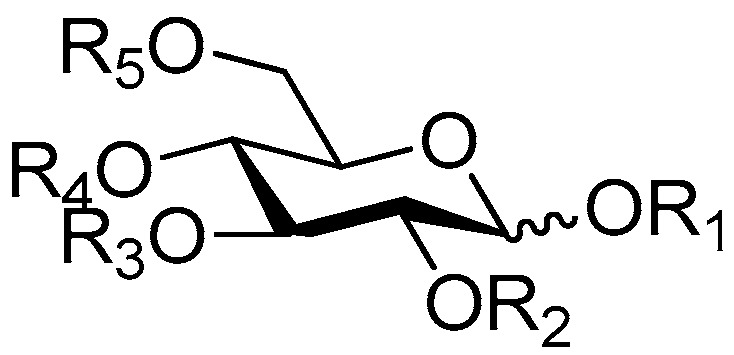
Structures of compounds **16**–**20**.

**Figure 5:**
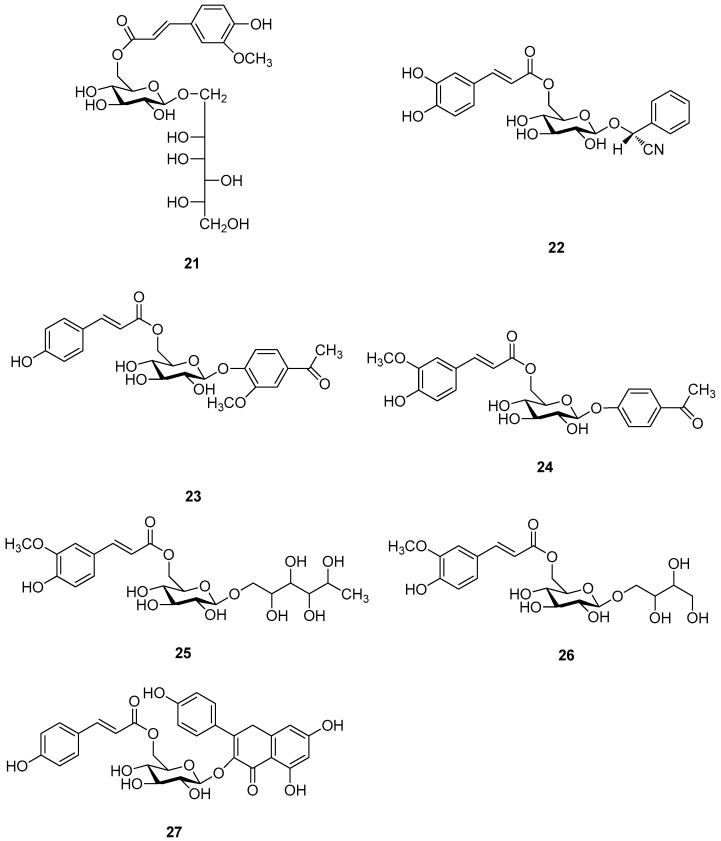
Structures of compounds **21**–**27**.

The 27 monosaccharide [16,18,20,21,22,23,24,25,26,27,28,29,30,31,32,33,34,35] esters **1**–**10**, **21**–**27** represent the simplest structures found among CASEDs (Figure 1, Figure 2, Figure 3, Figure 4 and Figure 5). The main structural moiety of these compounds (Figure 2 and Figure 5), is a β-d-glucose ester, or an α-l-rhamnose ester in compounds **11**–**15** (Figure 3). Compounds **16**–**20** exist as anomeric mixtures in solution and the phenylacrylic group is often attached at the C-6 position of the glycosyl moiety. Coincidentally, compounds **11**–**15** possess the same *p*-methoxy- cinnamoyl group attached to the rhamnose unit though an ester bond in the monosaccharide ester. Compounds **2**, **4** and **20** are phenylpropanol esters linked with glucose as the important part. Compounds **1** and **3** contain two different phenylpropanols attached to one glucose molecule. Ningposide D (**14**) [16] is also an anomeric mixture of rhamnose esters and the anomeric ration α/β is 3:1, here it was drawn as the α-l-rhamnose ester. Isolated from the underground parts of *Globularia orientali*, globularitol (**21**) has a carbohydrate chain moiety, formed by a glucitol group. It has the ability to efficiently scavenge free radicals [32]. Grayanin (**22**) has a mandelonitrile unit connected at the C-1 position in the glucose. This compound is a unique cyanogenic glycoside among CASEDs [20]. The benzeneacetonitrile group of grayanin may be originated from phenylalanine from the biosynthetic pathway viewpoint. Up to now, paederol A (**25**) and B (**26**), are the only two reported CASEDs with acyclic sugars. By the way, paederol A and B did not exhibit obviously cytotoxicity in the Lu1 (lung cancer), LNCaP (prostate cancer) and MCF-7 (breast cancer) [34]. Kaempferol 3-*O*-β-d-(6-*O*-*p*-*E*-coumaroyl)-glucopyranoside (**27**) is the only flavonoid of CASEDs, which possess inhibitory activity towards a drug-metabolizing enzyme, CYP3A4 [35].

### 2.2. Disaccharide Esters

**Figure 6:**
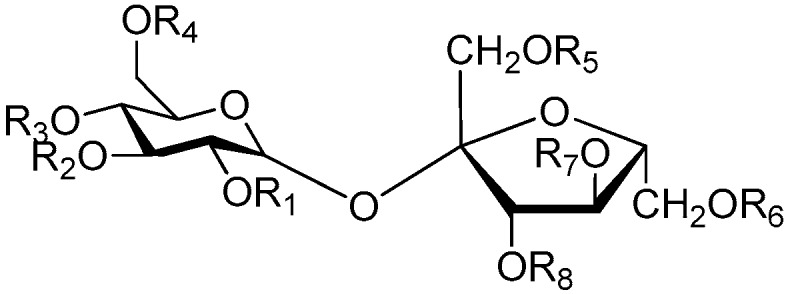
Structures of compounds **28**–**120**.

**Figure 7:**
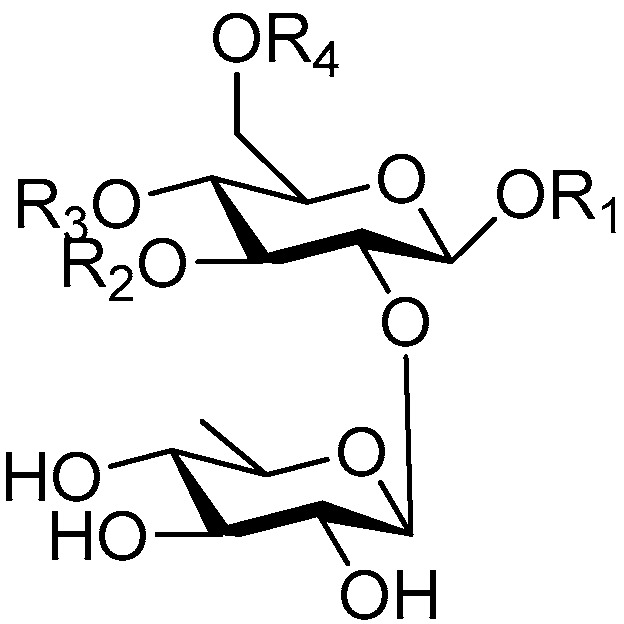
Structure of compound **121**.

**Figure 8:**
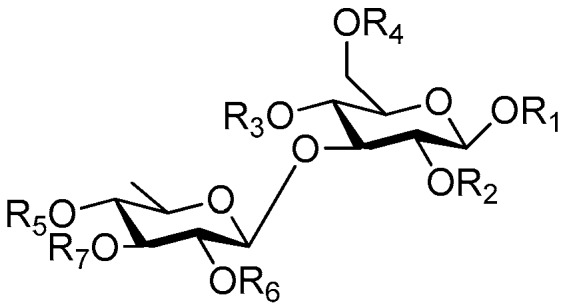
Structures of compounds **122**–**140**.

**Figure 9:**
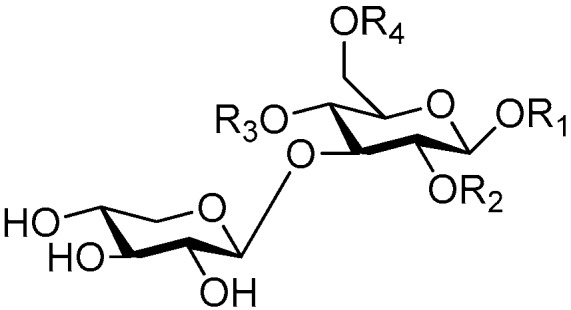
Structure of compound **141**.

**Figure 10:**
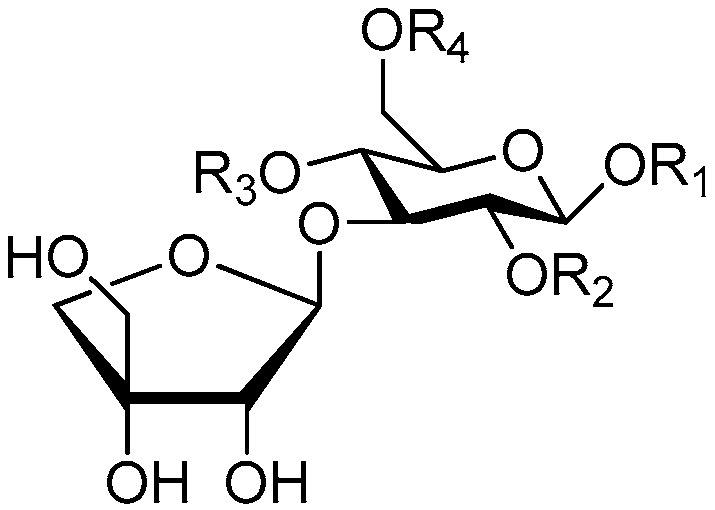
Structures of compounds **142**–**143**.

**Figure 11:**
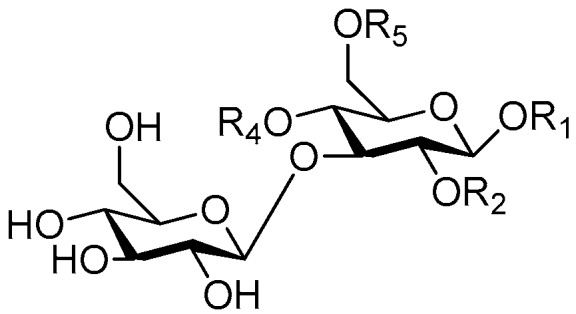
Structure of compound **144**.

**Figure 12:**
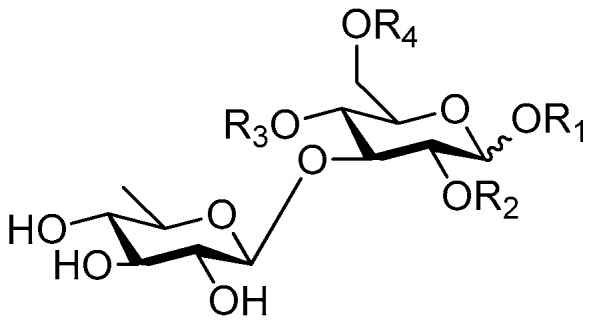
Structures of compounds **145**–**147**.

**Figure 13:**
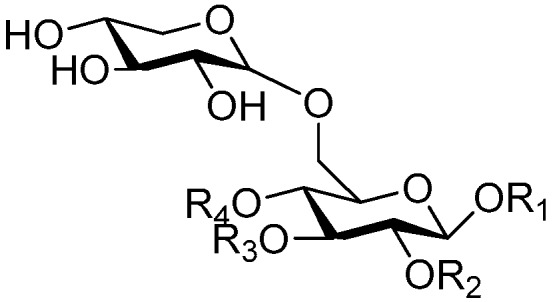
Structure of compound **148**.

**Figure 14:**
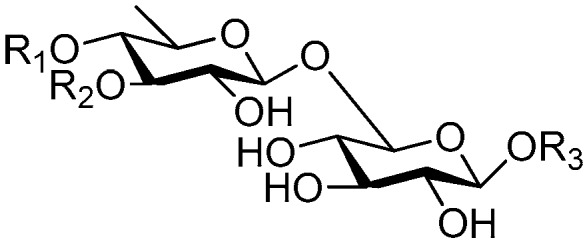
Structure of compound **149**.

**Figure 15:**
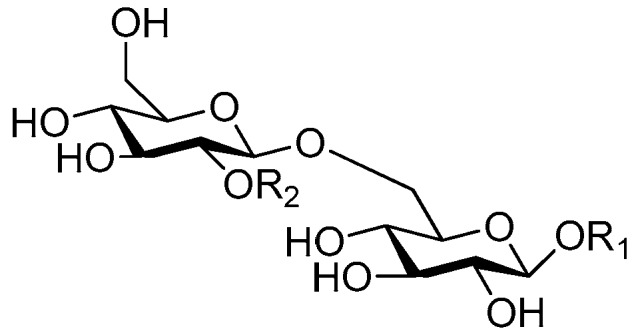
Structures of compounds **150**–**155**.

**Figure 16:**
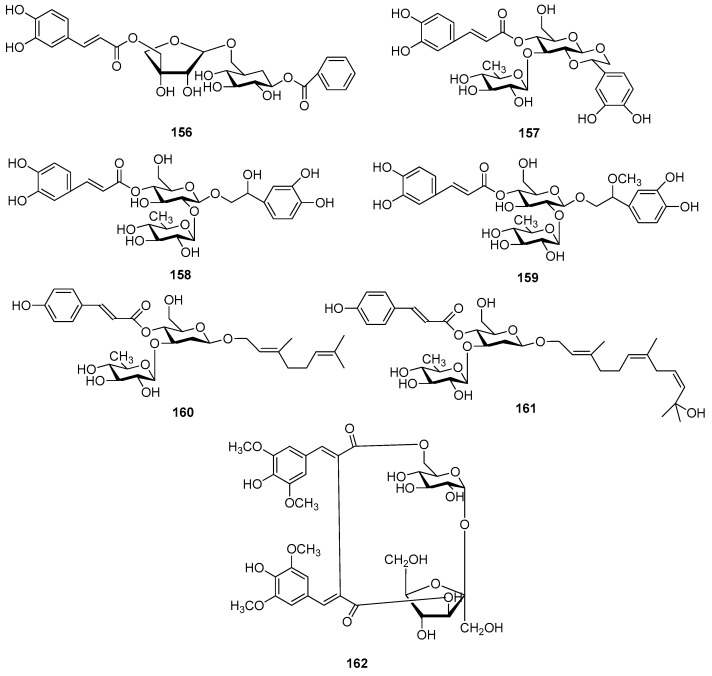
Structures of compounds **156**–**162**.

Disaccharide esters **28**–**162** (Figure 6, Figure 7, Figure 8, Figure 9, Figure 10, Figure 11, Figure 12, Figure 13, Figure 14, Figure 15 and Figure 16) [4,5,10,13,15,17,19,24,25,29,36,37,38,39,40,41,42,43,44,45,46,47,48,49,50,51,52,53,54,55,56,57,58,59,60,61,62,63,64,65,66,67,68,69,70,71,72,73,74,75,76,77,78,79,80,81,82,83,84,85,86,87,88,89,90,91] constitute the largest group among CASEDs. Their glycosyl parts include glycosyl groups, with glucopyrannosyl, rhamnopyranosyl, fructofuranosyl, and arabinopyranosyl ones being the most important and sucrose units as found in compounds **28**–**120**, **162** are more rare,. Among them, the glycosyl unit in **28**–**120** has the anomeric carbon on α-d-glucose linked to a β-d-fructose. The ester bond is often formed at the C-6 position of α-d-glucose and C-3 position of β-d-fructose. The compounds **122**–**140** are composed of α-l-rhamnose and β-d-glucose, with a connection between the C-1 location of α-l-rhamnose and C-3 position of β-d-glucose. The cinnamic acid unit is mainly connected to the C-4 position of β-d-glucose, and less often in the C-6 location. The glycosyl moieties of compounds **145**–**147** are similar to those of **122**–**140**, and the configuration of the hydroxyl attached to the anomeric carbon of glucose could not be determined. The aglycone part of compounds **150**–**155** is two β-d-glucoses joined by C-1 and C-6, and the functional group is attached to the C-2 position of the parent nucleus.

Sibiricose A5 (**28**), tenuifoliside A (**51**) and DISS (**73**) from the root of *Polygala sibirica* (Yuanzhi) [13], have the same core sucrose unit and the ester is always connected at the C-6 position of α-d-glucose and C-3 position of β-d-fructose. These compounds have anti-depression properties. In 1968, verbascoside (=acteoside **131**) was the first CASED isolated from the medical plant *Syringa vulgaris* (*Oleaceae*) [3]. So far, it has been reported in nine families. Magnoloside A (**121**) from medicinal plants of the *Magnoliaceae* family is unique among the phenylpropanoids in rarely occurring alone as the core glycosyl [62]. In addition, crenatoside (**157**) has a novel annular framework which attaches the C-1 and C-2 of the glucose to a hexatomic oxygen ring [91]. Glomeratose E (**162**) possesses a (*E*,*E*)-β,β′-*bis*-sinapoyl group between the α-d-glucose and β-d-fructose [38].

### 2.3. Trisaccharide Esters

**Figure 17:**
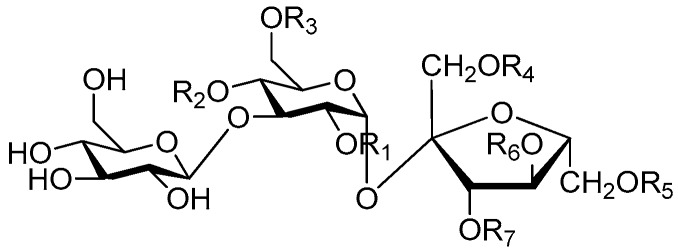
Structures of compounds **163**–**166**.

**Figure 18:**
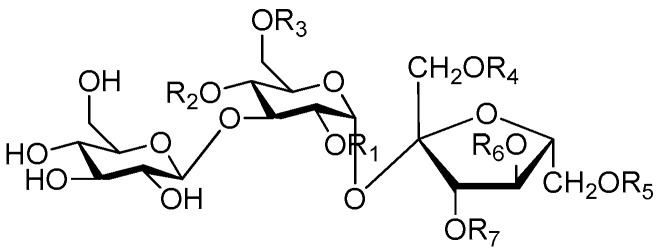
Structures of compounds **167**–**168**.

**Figure 19:**
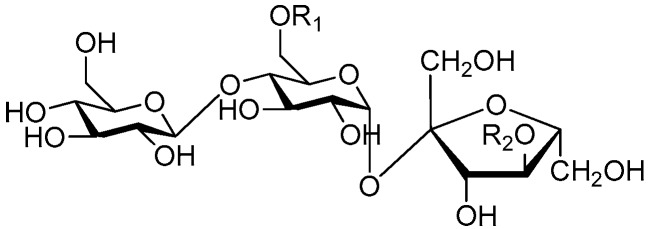
Structures of compounds **169**–**172**.

**Figure 20:**
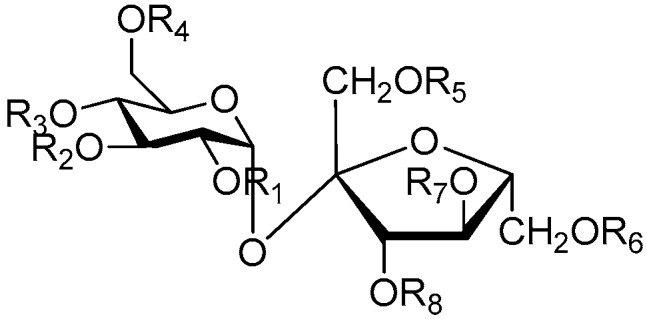
Structures of compounds **173**–**174**.

**Figure 21:**
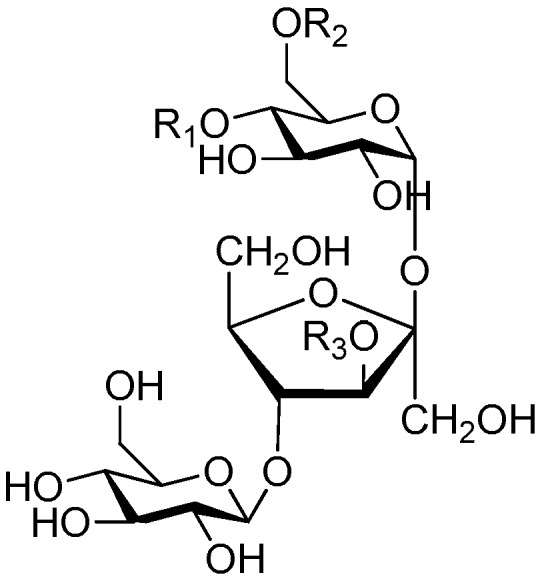
Structure of compound **175**.

**Figure 22:**
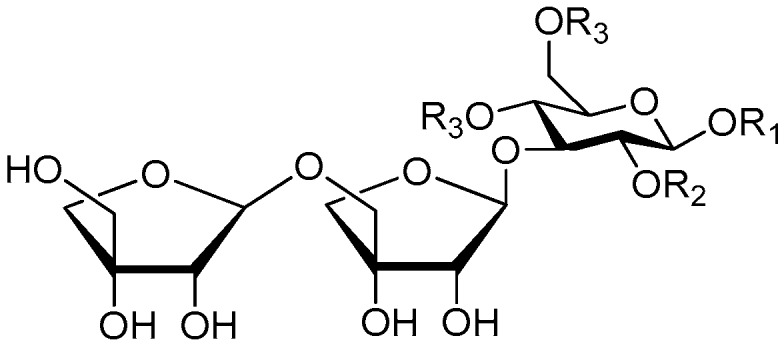
Structures of compounds **176**–**177**.

**Figure 23:**
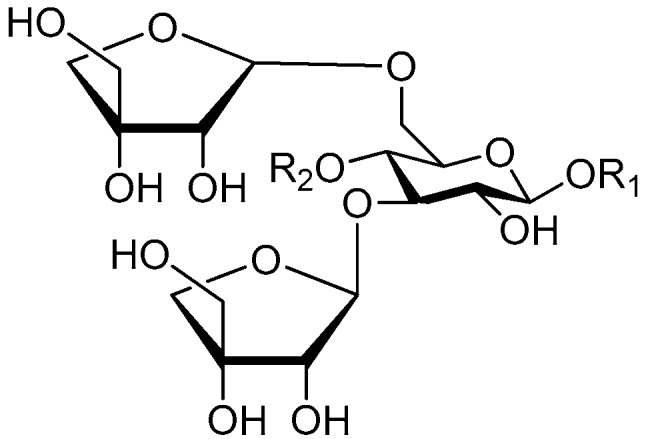
Structure of compound **178**.

**Figure 24:**
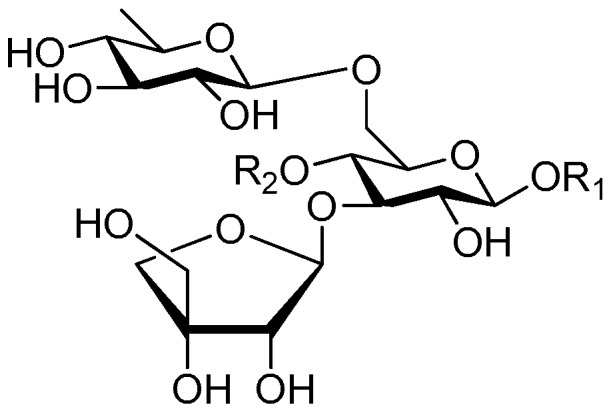
Structures of compounds **179**–**182**.

**Figure 25:**
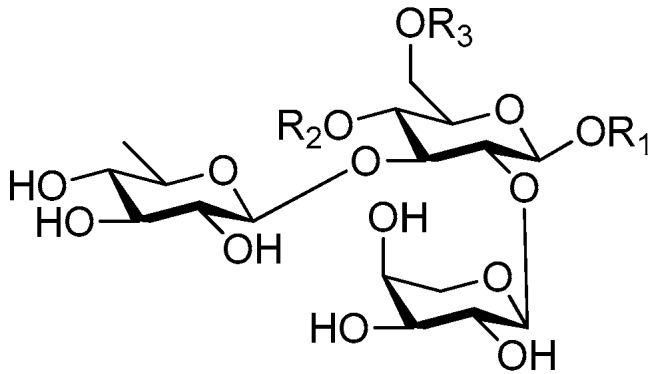
Structures of compounds **183**–**187**.

**Figure 26:**
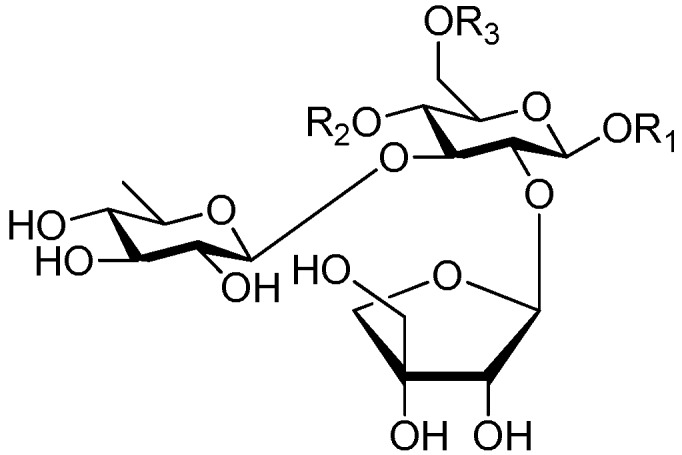
Structures of compounds **188**–**192**.

**Figure 27:**
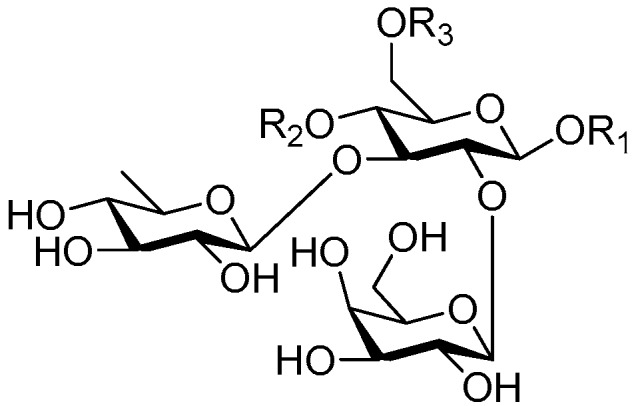
Structure of compound **193**.

**Figure 28:**
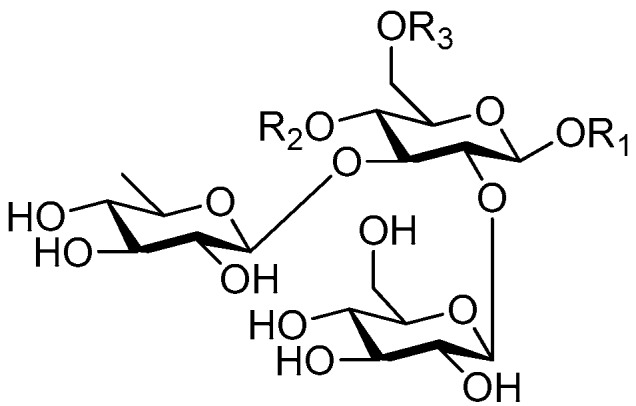
Structures of compounds **194**–**195**.

**Figure 29:**
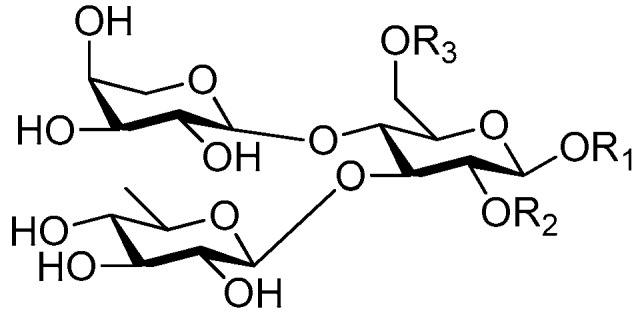
Structure of compound **196**.

**Figure 30:**
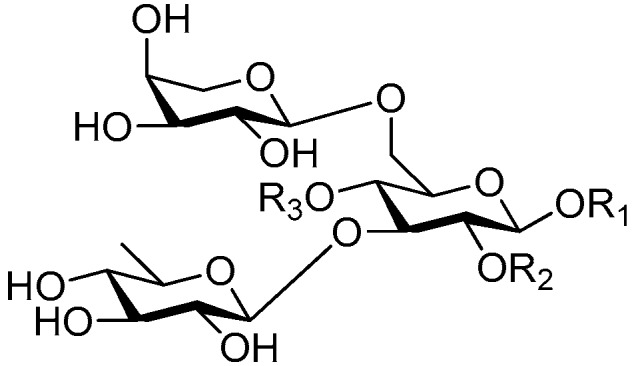
Structures of compounds **197**–**201**.

**Figure 31:**
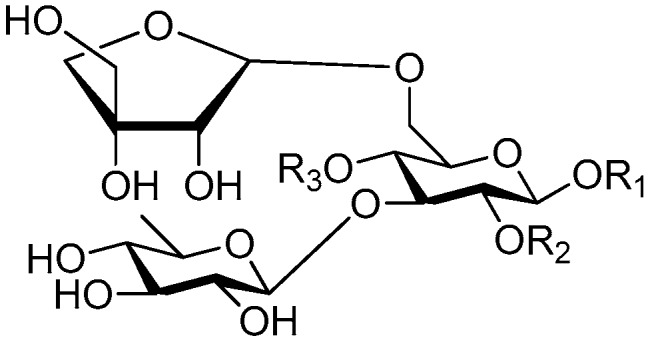
Structures of compounds 2**02**–**205**.

**Figure 32:**
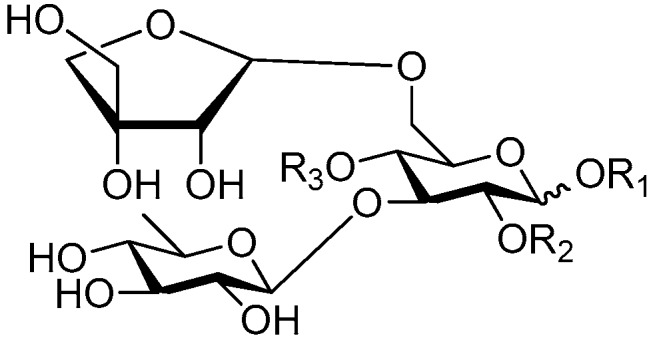
Structures of compounds **206**–**207**.

**Figure 33:**
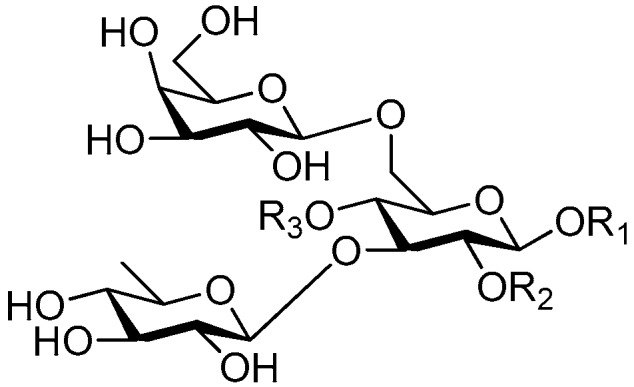
Structures of compounds **208**–**213**.

**Figure 34:**
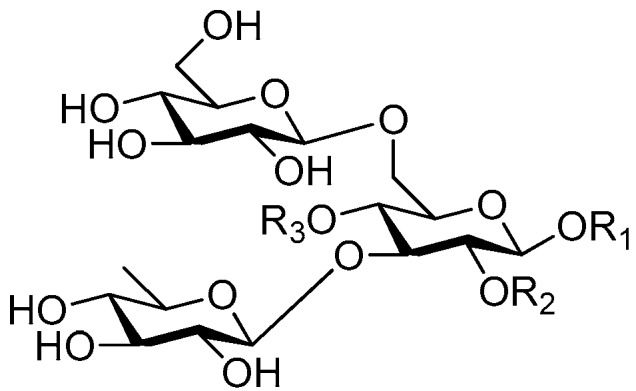
Structures of compounds **214**–**216**.

**Figure 35:**
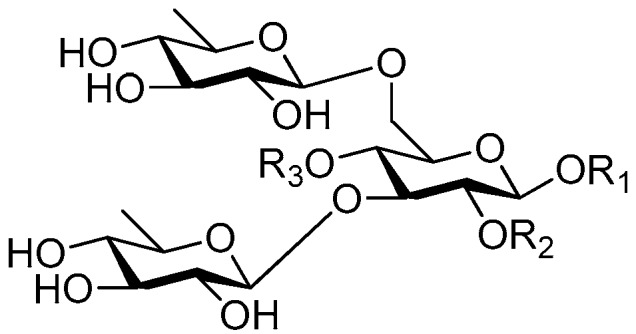
Structures of compounds **217**–**218**.

**Figure 36:**
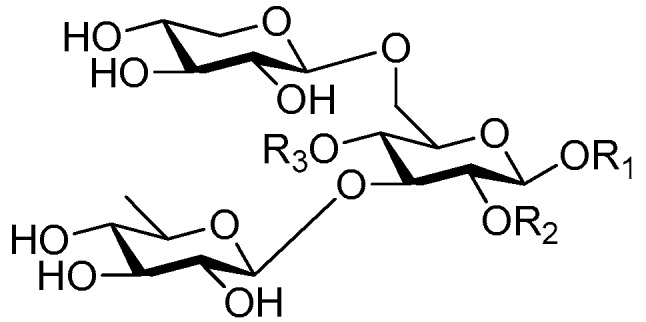
Structure of compound **219**.

**Figure 37:**
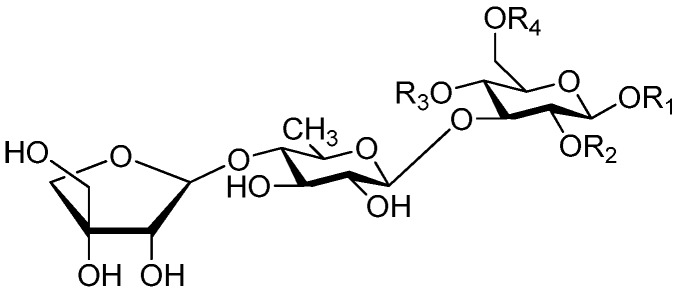
Structure of compound **220**.

**Figure 38:**
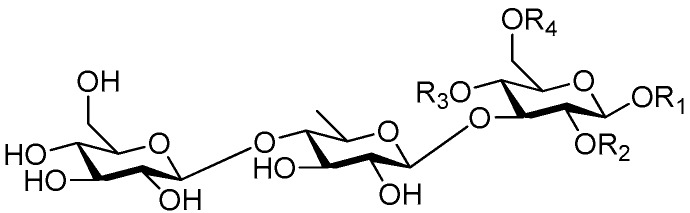
Structures of compounds **221**–**223**.

**Figure 39:**
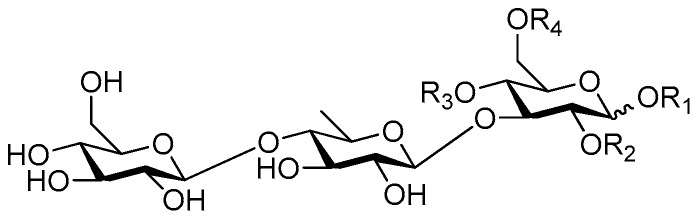
Structure of compound **224**.

**Figure 40:**
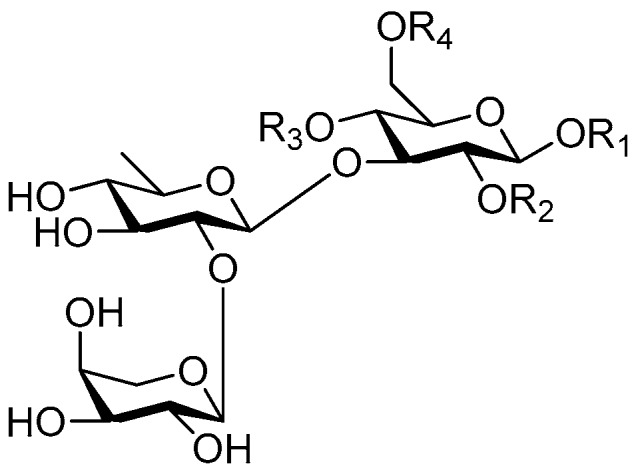
Structures of compounds **225**–**227**.

**Figure 41:**
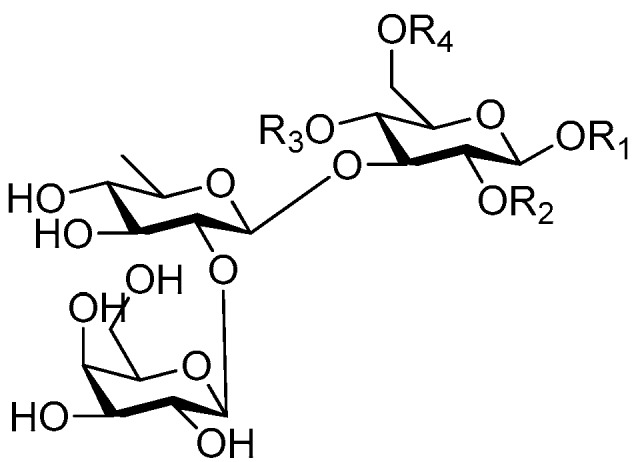
Structure of compound **228**.

**Figure 42:**
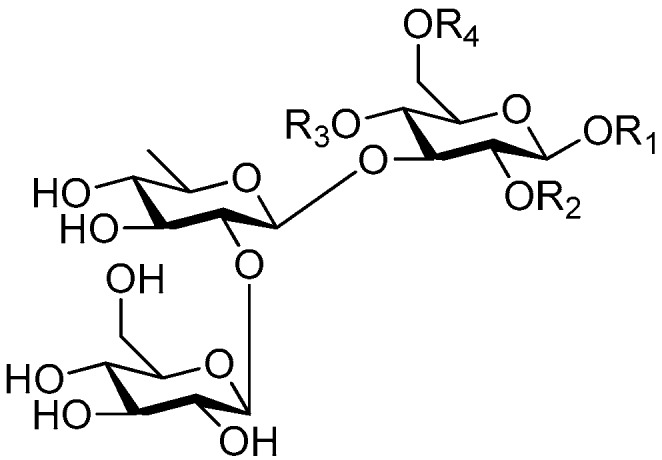
Structure of compound **229**.

**Figure 43:**
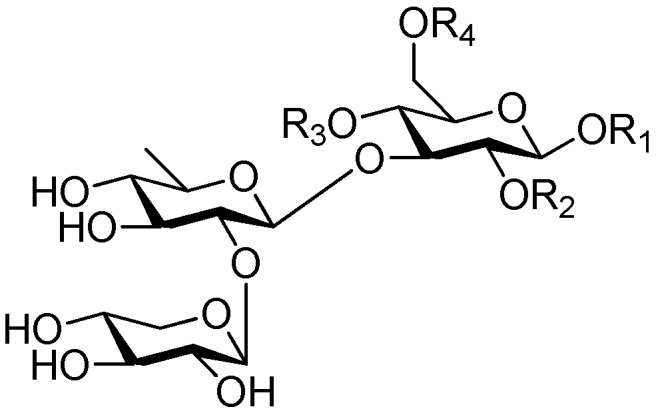
Structure of compound **230.**

**Figure 44:**
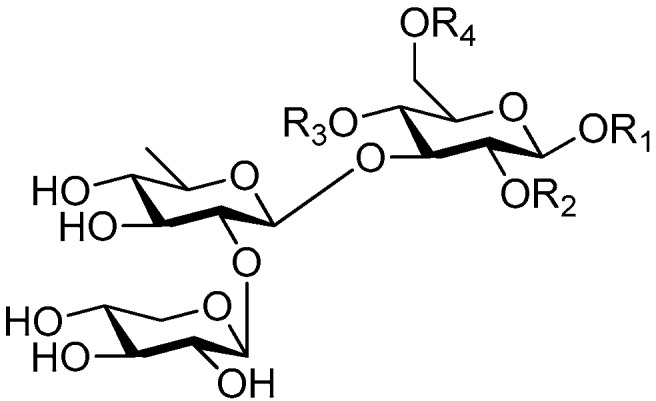
Structures of compounds **231**–**232**.

**Figure 45:**
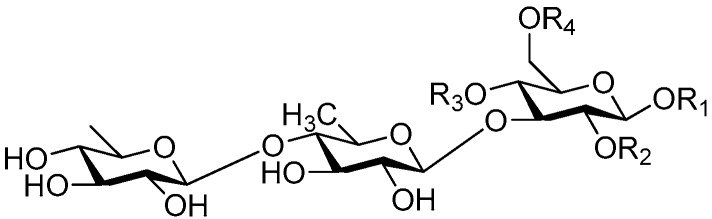
Structures of compounds **233**–**234.**

**Figure 46:**
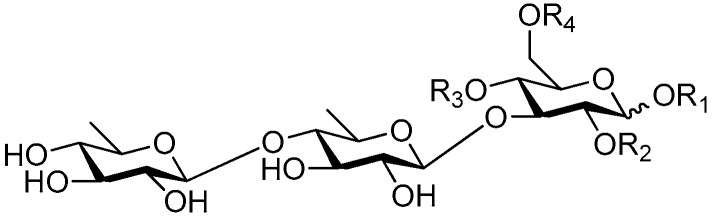
Structures of compounds **235**–**238**.

**Figure 47:**
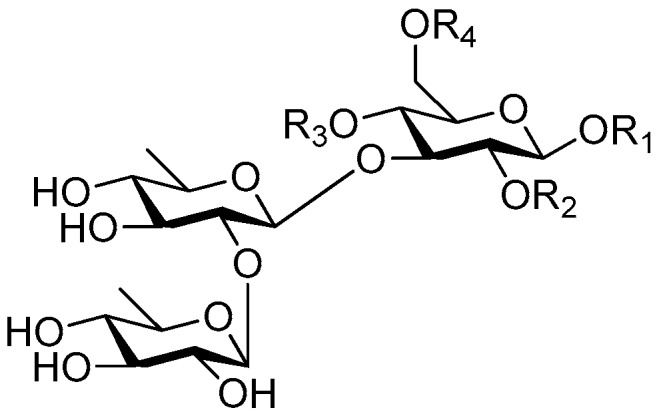
Structures of compounds **239**–**240**.

**Figure 48:**
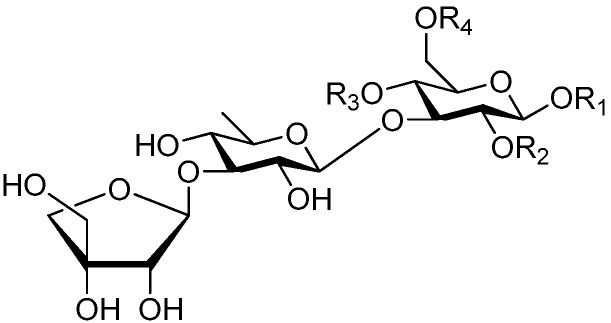
Structures of compounds **241**–**242**.

**Figure 49:**
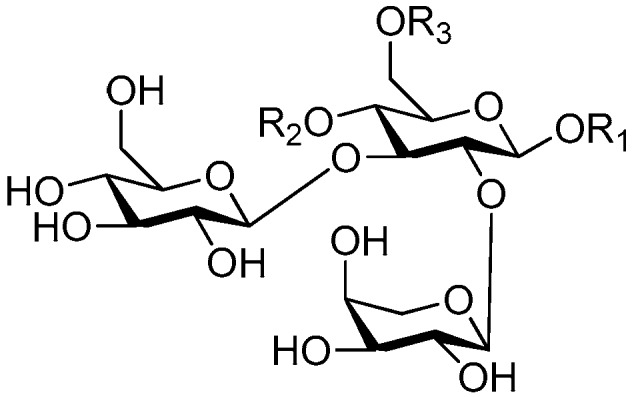
Structure of compound **243**.

**Figure 50:**
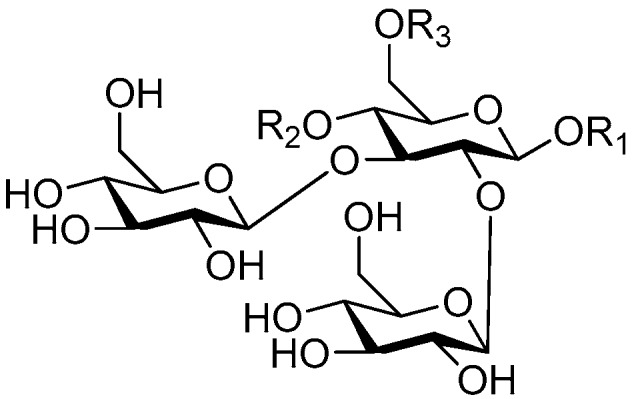
Structure of compound **244**.

**Figure 51:**
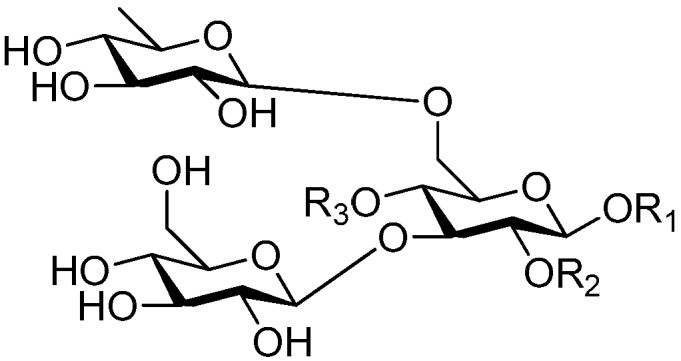
Structure of compound **245**.

**Figure 52:**
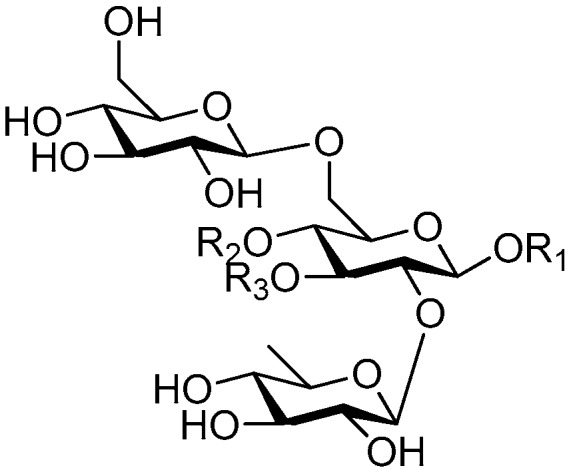
Structure of compound **246**.

**Figure 53:**
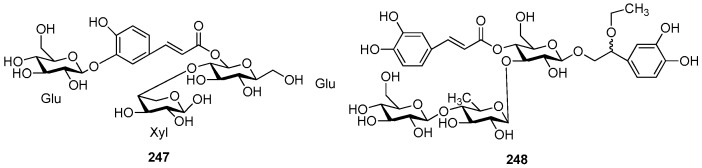
Structures of compounds **247**–**248**.

**Figure 54:**
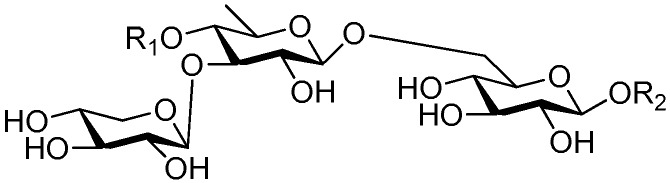
Structures of compounds **249**–**254**.

Ninety three compounds **163**–**254** [5,19,21,24,37,46,51,55,63,64,65,66,67,75,78,79,80,81,82,83,84,85,86,93,94,95,96,97,98,99,100,101,102,103,104] represent the trisaccharide ester category. They are mainly obtained from the *Scrophulariaccae* plant family. The most common glycosyl moieties are sucrose, with glucose as core unit (compounds **163**–**174**, **175**, Figure 17, Figure 18, Figure 19 and Figure 20 and Figure 21), di-apiose combined with glucose (**176**–**178**, Figure 22 and Figure 23), glucose as the kernel glycosyl (**179**–**246**, **247**–**248**, Figure 24, Figure 25, Figure 26, Figure 27, Figure 28, Figure 29, Figure 30, Figure 31, Figure 32, Figure 33, Figure 34, Figure 35, Figure 36, Figure 37, Figure 38, Figure 39, Figure 40, Figure 41, Figure 42, Figure 43, Figure 44, Figure 45, Figure 46, Figure 47, Figure 48, Figure 49, Figure 50, Figure 51 and Figure 52 and Figure 53), and rhamnose as the central part with its terminal carbon combined with glucose and the C-3 connected with xylose (**249**–**254**, Figure 54). The phenylpropanoid groups usually esterify the C-3 and C-4 positions of fructose, C-3, C-4 and C-6 of glucose and C-4 of rhamnose. Tricornoses E (**165**) and F (**166**) from the *Polygalaceae* family possess two different phenylpropanoids attached to one fructose molecule [37]. Lilongiside (**173**), reiniose D (**174**) and hydrangeifolin II (**253**) differ from other trisaccharide esters in that their three sugar cores are not combined as a whole chain [21,46,55]. The aglycone groups of lilongiside and reiniose D are sucrose with glucose, rhamnose. Hydrangeifolin II is composed of caffeoyl glycoside with a diglycosyl unit esterified with an ester linkage. This compound has a weak DPPH free radical scavenging activity. Teucrioside (**229**) from the *Labiatae* family is the only CASED that has a lyxose moiety, rarely occuring in higher plants [101]. The anomeric carbon configuration of glucose unit in ligupurpuroside F (**234**) is not determined [24]. Rossicaside F (**254**) exists as epimers at the β-C of the phenethyl alcohol moiety (*R*,*S*-β-*O*Et) [98].

### 2.4. Tetrasaccharide Esters

**Figure 55:**
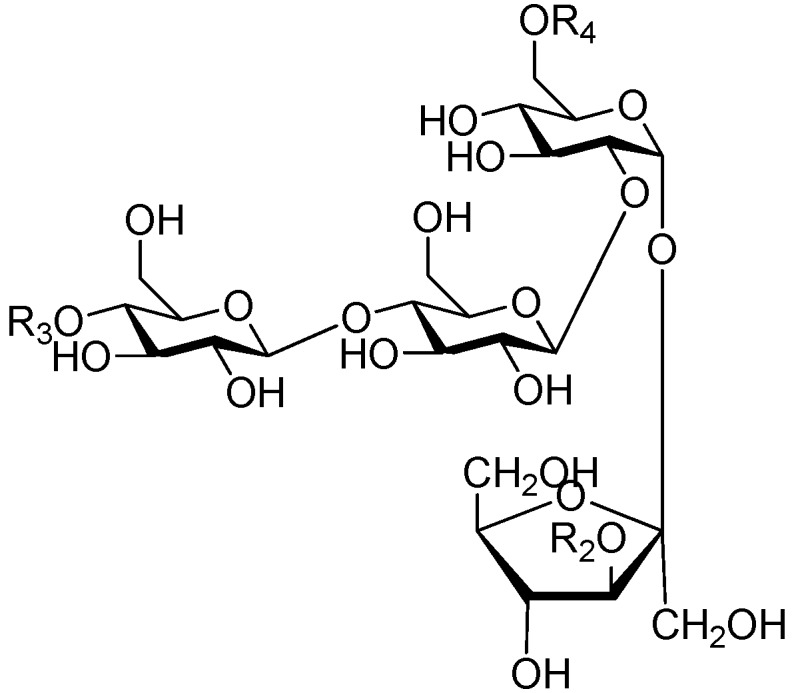
Structures of compounds **255**–**260**.

**Figure 56:**
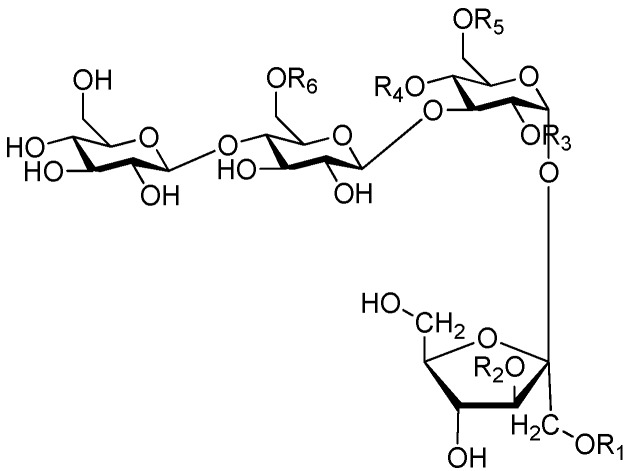
Structure of compound **261**.

**Figure 57:**
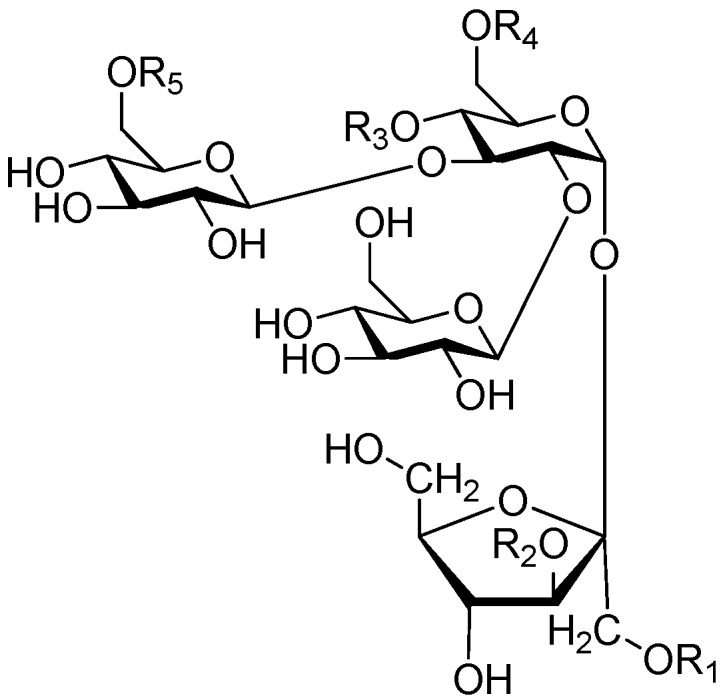
Structures of compounds **262**–**274**.

**Figure 58:**
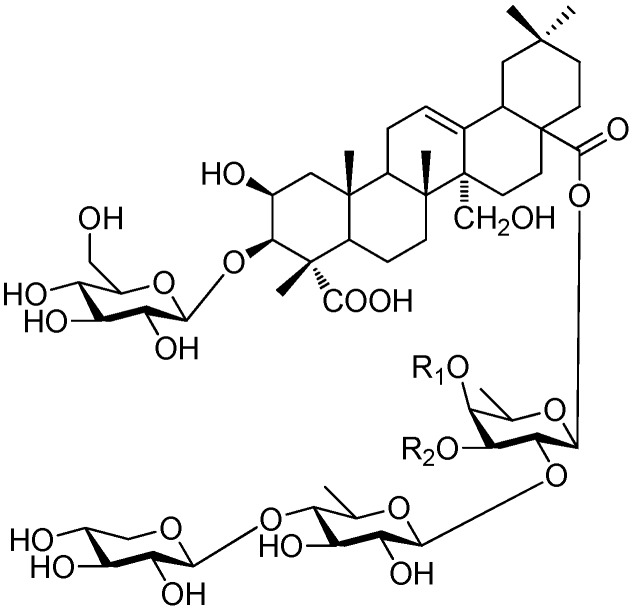
Structure of compound **275**.

**Figure 59:**
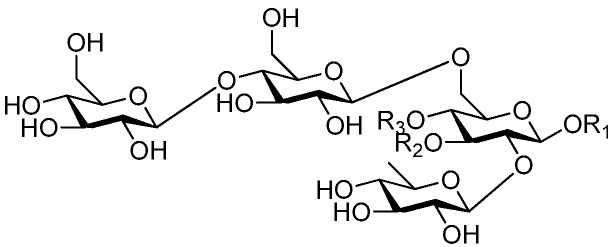
Structure of compound **276**.

**Figure 60:**
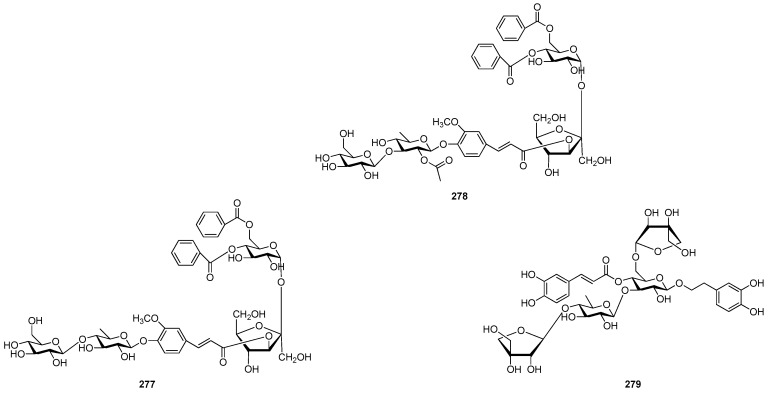
Structures of compounds **277**–**279**.

Of all the tetrasaccharide esters **255**–**279** (Figure 55, Figure 56, Figure 57, Figure 58, Figure 59 and Figure 60) [37,38,46,62,92,93,105,106,107], 23 are found in *Polygalaceae* plants Most of the phenylacrylic moieties are coumaroyl, feruloyl and sinapoyl groups. According to the core glycosyl type, these compounds can be classified into four groups, including the combination of fructose with three glucoses (**255**–**274**, Figure 55, Figure 56 and Figure 57), rhamnose, fructose with two glucoses (**277**–**278**, Figure 60), the other tetrasaccharide esters (**275**, **276**, **279**, Figure 58, Figure 59 and Figure 60). Senegoses F–I (**261**, **272**–**274**) [105], whose absolute configurations were established by spectroscopic and chemical means, were purified from *Polygala senega* var. *latifolia* To_RR_. *et* G_RAY_(Polygalaceae). Polygalasaponin XLII (**275**) which was obtained from the roots of *Polygalaglomerata* Lour belongs to the oleanane-type saponins, [107]. Its fucose C-4 position attaches to a 3,4-dimethoxycinnamoyl by an ester bond. The structures of fallaxose A (**277**) and fallaxose B (**278**), found in the roots of *Polygala fallax*, are similar, except for the acetyl group and the glucose location. Both are esterified with ferulic acid [92].

### 2.5. Other Sugar Esters

**Figure 61:**
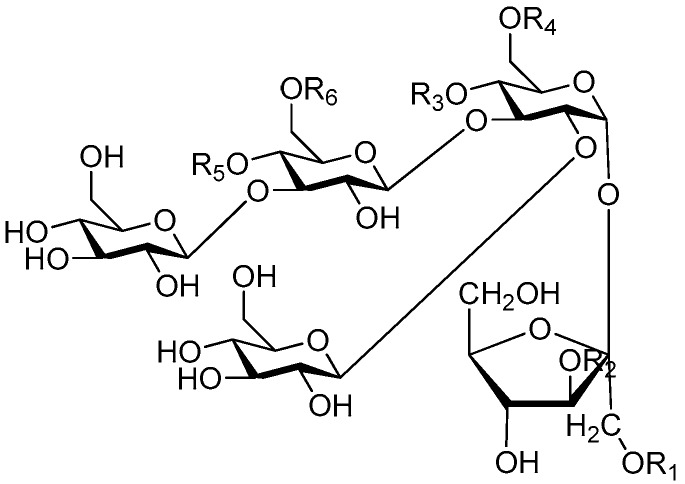
Structures of compounds **280**–**294**.

**Figure 62:**
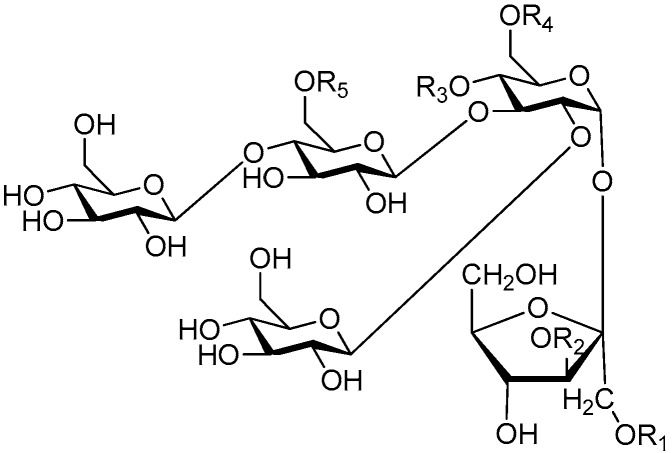
Structures of compounds **295**–**305**.

**Figure 63:**
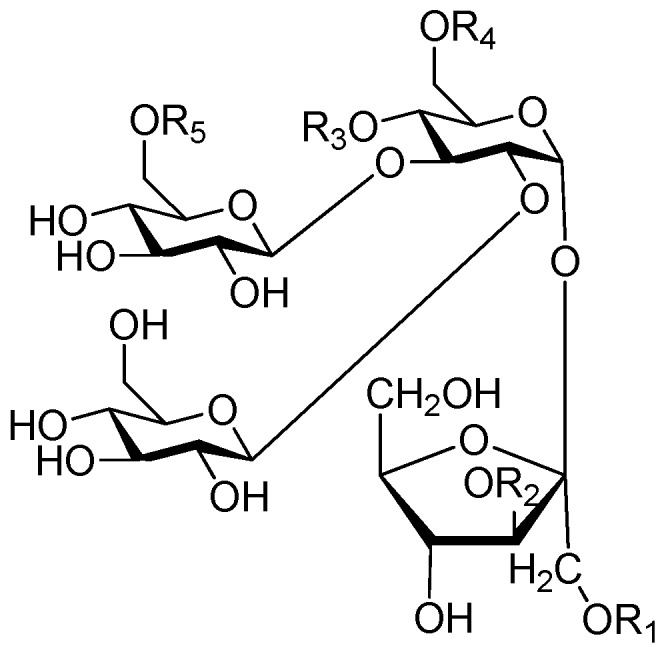
Structures of compounds **306**–**316**.

**Figure 64:**
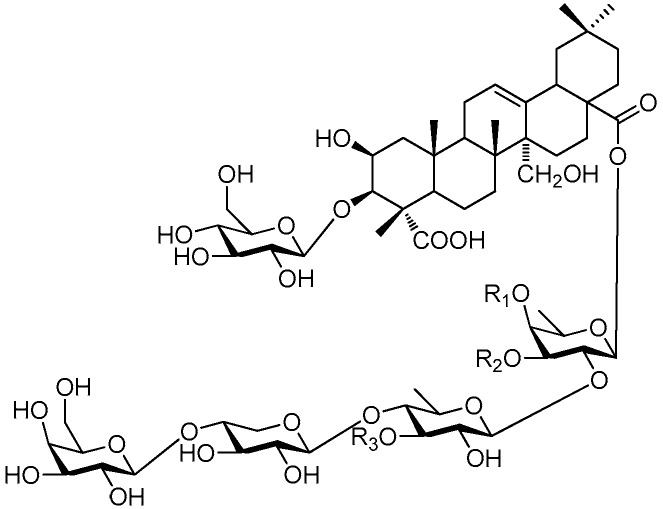
Structures of compounds **317**–**320**.

**Figure 65:**
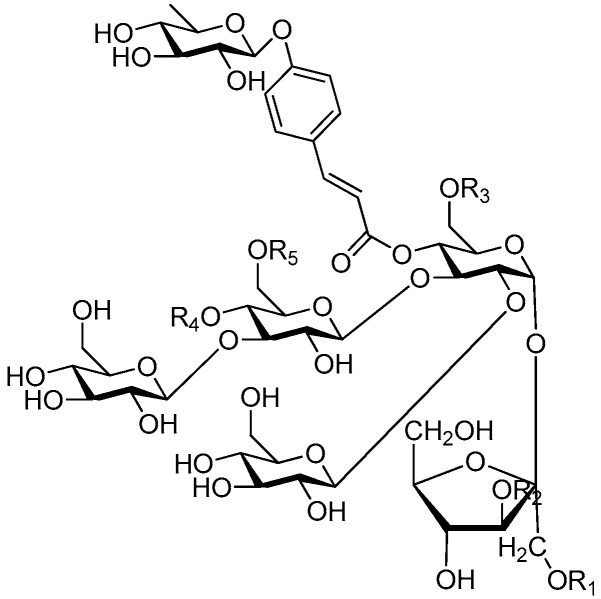
Structures of compounds **321**–**322**.

**Figure 66:**
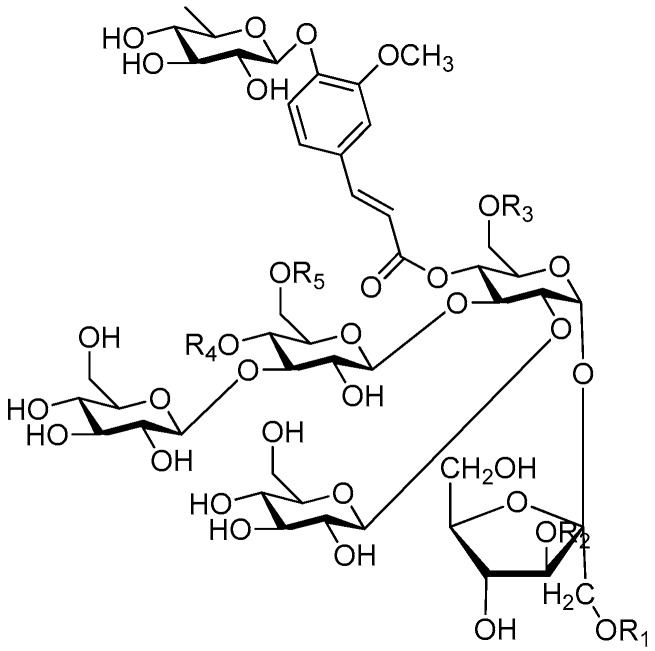
Structures of compounds **323**–**324**.

**Figure 67:**
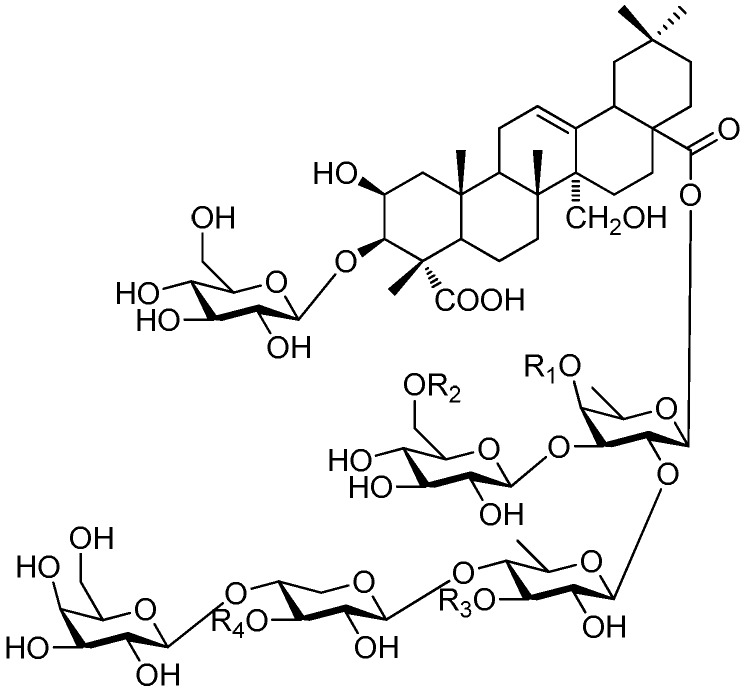
Structures of compounds **325**–**328**.

**Figure 68:**
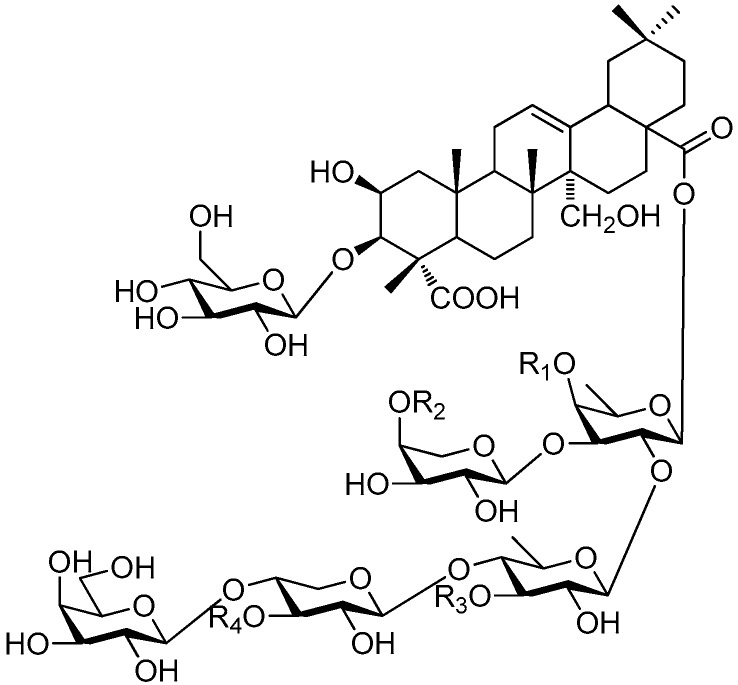
Structure of compound **329**.

**Figure 69:**
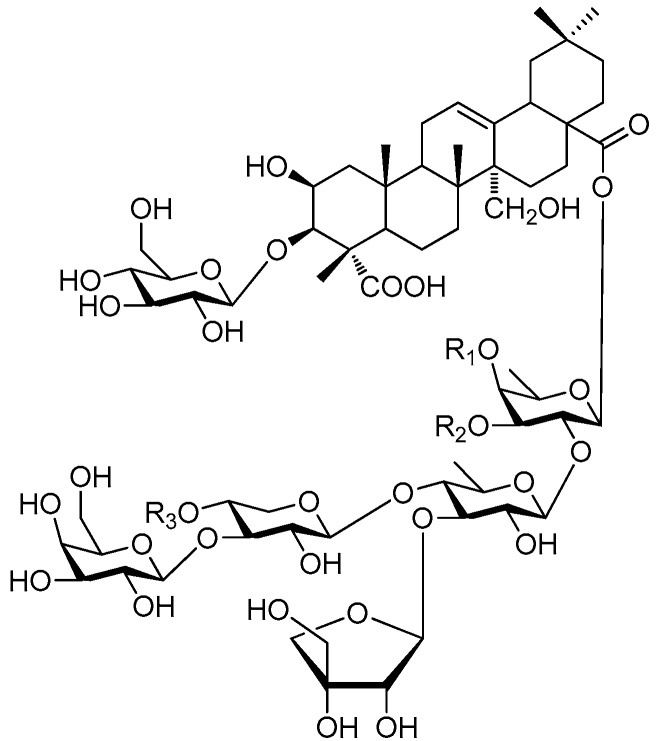
Structure of compound **330**.

**Figure 70:**
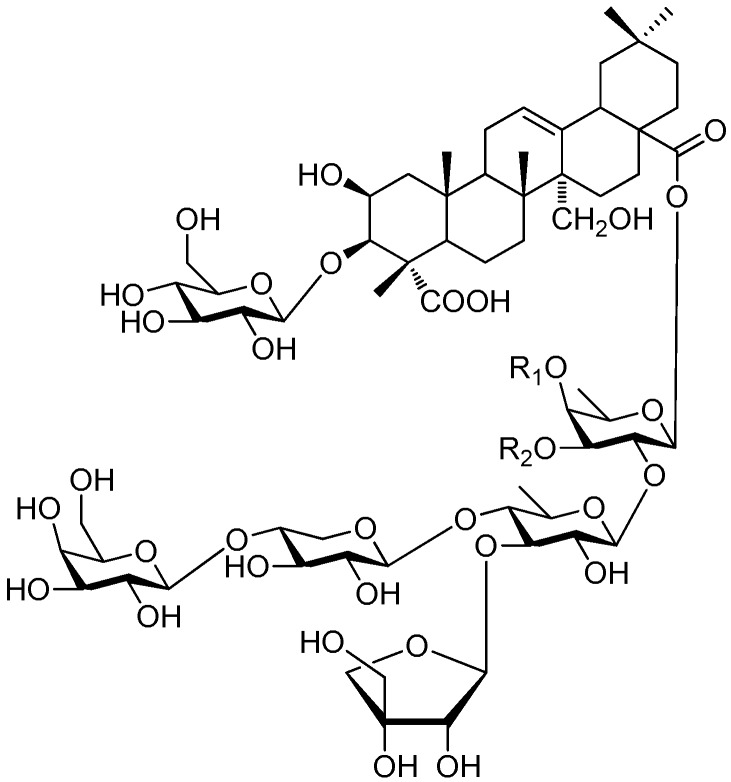
Structures of compounds **331**–**332**.

**Figure 71:**
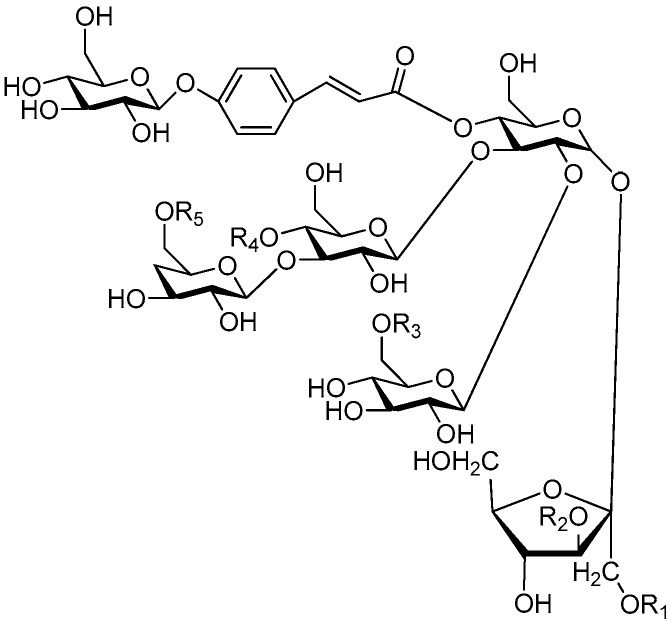
Structure of compound **333**.

**Figure 72:**
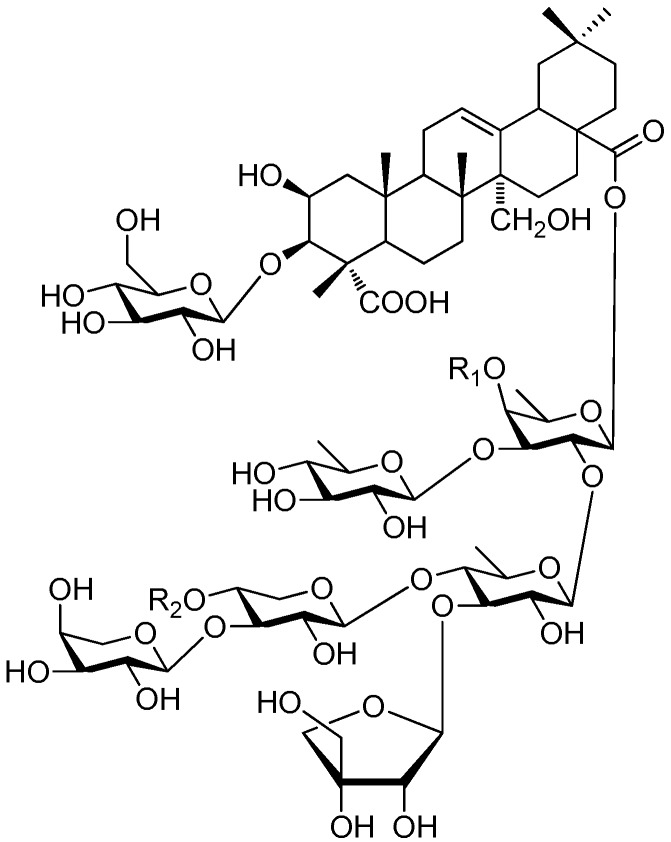
Structure of compound **334**.

To our knowledge, pentasaccharide esters **280**–**320** (Figure 61, Figure 62, Figure 63 and Figure 64) [11,12,46,92,93,107,108,109,110], hexsaccharide esters **321**–**333** (Figure 65, Figure 66, Figure 67, Figure 68, Figure 69, Figure 70 and Figure 71) [11,12,56,107,109,110], heptasaccharide esters **334** (Figure 72) [56] were all found in the *Polygalaceae* family and most of them form a series of similar type compounds. That is to say, CASEDs with higher carbon numbers are rarely found in plants outside the *Polygalaceae*. The phenylacrylic groups usually locate at C-1 of fructose, C-4 of glucose, as well as C-4 of fucose. Most glycosyl moieties of pentasaccharide esters are four glucoses and a fructose with different locations and sequence. Tenuifolioses A and B (**285**, **288**), obtained from *Polygala tenuifolia* Willd, showed neuroprotective activity. Tenuifolioses A and B have the same glycosyl core, with β-d-glucoses connected at the C-1 and C-4 position and the first glucose combined with another glucose at C-2 and β-d-fructose at C-1 [12]. Compounds **280**–**294** with this same sugar core serve to remind researchers of the need for more studies on these compounds to find more precursor compounds of anti-depression drugs. The tenuifoliose A–E (**284**–**288**), senegose A–E (**301**–**305**), J–O (**295**–**300**) type of oligosaccharide multi-esters are esterified with coumaric and ferulic acids [108,111]. Compounds **306**–**307**, **311** [93] are pentasaccharide esters having the same glycosyl connection sequence as that of reiniose G (**265**) and have a *p*-coumaroyl residue at C-6 of glucose [38]. Compounds **308**–**310**, **312**–**316** are also pentasaccharide esters, but with a feruloyl residue at C-6 of glucose. Compounds **319**–**320** and **325**–**330** are CASEDs belonging to the oleanane-type saponins and found in the root parts of *Polygala glomerata* Lour [107], which have the same parent nuclei as polygalasaponin XLII (**275**). To our knowledge, only one heptasaccharide ester (polygalasaponin XXXII, **334**) was reported, and it is also an oleanane-type saponin, with hippocampus-dependent learning and memory enhancing activity. Polygalasaponin XXXII [56], as the representative of oleanane-type saponins in CASEDs, has also captured attention of researchers to do more investigation on the other compounds of the class (**317**–**320**, **325**–**332**) in order to identify compounds with the same activity or with more sugars that might improve the improve hippocampus-dependent learning and memory enhancing activity of polygalasaponin XXXII.

## 3. Biological Activities

To date, approximately 334 CASEDs have been isolated from various medicinal plants and their structures characterized. However, the biological activities, mechanism of action and structure-activity-relationships (SAR) of many CASEDs have rarely been explored up to now. Hence, an overview of the pharmacological activities of the CASED may serve as valuable indication to further probe into their full therapeutic potentials.

### 3.1. Anti-Depression Activity and Neuroprotective Activity

Depression, one of the major mental disorders, is accompanied by symptoms such as emotional slump, reduced physical activities, feelings of helplessness and pessimism and even suicide attempts. At present there are three main points of view regarding the pathogenesis of depression, including the biogenic amine theory, the nerve nutrition theory and the cytokines theory.

Sibiricose A5 (**28**), tenuifoliside A (**51**), 3′,6-disinapoylsucrose (DISS, **73**), tenuifoliside B (**52**), buergerisides A_1_ (**13**), B_1_ (**12**), B_2_ (**15**) and C_1_ (**11**), tenuifolioses A (**285**) and B (**288**) show obvious antidepressant activity [10,12,13,18]. Sibiricose A5 (**28**) and tenuifoliside A (**51**), extracted from Chinese herbal medicine *Polygala tenuifolia* Willd, were found to dramatically protect PC12 cells damaged by glutamate [9]. Tenuifolioses A (**285**) and B (**288**) showed neuroprotective activity against glutamate and serum deficiency at a concentration of 1 × 10^−5^ mol·L^−1^ [12]. Liu et al. [1] discovered that DISS and tenuifoliside A (TEA, **51**), isolated from *Radix Polygalae*, showed protective effects on SH-SY5Y against Cort-induced injury. A study by Ikeya et al. [112] showed that tenuifoliside B (**52**) improved the scopolamine-induced impairment of passive avoidance response by promoting the cholinergic system. Buergerisides A_1_ (**13**), B_1_ (**12**), B_2_ (**15**) and C_1_ (**11**) from the roots of *Scrophularia buergeriana* exhibit protective activity on primary cultures of rat cortical cells after exposure to excitotoxin, glutamate according to an investigation by Kim et al. [18].

Further findings demonstrate that a possible mechanism of the antidepressant action of DISS maybe be related with hippocampal neuroplasticity and neuroproliferation. DISS possesess potent and rapid antidepressant activity, which are mediated via brain MAO-A and MAO-B activity and upregulated serum cortisol levels induced by CMS [113]. In neuronal cells, DISS-mediated regulation of BDNF gene expression is associated with CREB-mediated transcription of BDNF upstream activation of ERK1/2 and CaMKII to cause neuroprotective and antidepressant effects [114]. Dong et al. [8] discovered that the neurotrophic mechanism of TEA (**b24**) in C6 cells correlates with TrkB/BDNF/ERK and TrkB/BDNF/PI3K.

### 3.2. Anticancer Activity

Belonging to the family of serine/threonine protein kinases that are activated by Ca^2+^, Protein Kinase C (PKC) is involved in signal transduction, and cellular proliferation and differentiation. It also plays an important role in cell cycle control, tumor genesis, antitumor drug resistance and apoptosis. PKC has been proved to be related with the activation of HIV-1 gene expression, tumor promotion, and the inhibition of apoptosis in leukemia cells. Therefore, it makes a lot of sense to find chemical compounds from natural plants to inhibit the activity of PKC [50,54].

Takasaki et al. found that vanicoside A (**102**) and vanicoside B (**67**) from *Polygonum* *pensylvanirum* inhibited PKC activity with IC_50_ values of 44 μg/mL and 31 μg/mL, respectively [54]. After this preliminary work, LaVerne et al. [50] continued the isolation work on this plant in order to obtain possible homologues via HPLC-MS and isolated vanicosides C-F (**104**, **57**, **113**, **91**). Regretfully, LaVeme did not to do much research on the pharmacological activity of the vanicosides. Notably, acteoside (=verbascoside, **131**) from *Lantana camara* also shows PKC inhibitory activity in the rat brain with an IC_50_ of 25 μM [29]. With the widest distribution in the plant kingdom, acteoside has been widely applied to treat diseases such as cancer, inflammation, or immune disorders.

In the virus family, the Epstein-Barr virus (EBV) is a type of herpes virus causing cancer. EBV has been considered one of the causes of many kinds of malignant tumors such as nasopharyngeal carcinoma. EBV infection mainly occurs human oropharyngeal epithelial cells and B lymphocytes. Lapathoside A (**63**), lapathoside D (**31**), vanicoside B (**67**) and hydropiperoside (**37**) exhibit remarkable inhibitory effects on the EBV, which is early antigen induced by tumor-promoters, so it makes sense to focus on these four compounds as worthy anti-tumor-promoters for cancer chemoprevention [2,39].

Meanwhile, Takasaki et al. [39] reported that lapathoside A (**63**) and vanicoside B (**67**) inhibited two-stage carcinogenesis induced by 12-*O*-tetradecanoylphorbol-13-acetate (TPA). Moreover, vanicoside B exhibits remarkable inhibitory effects, which are initiated with a NO (nitric oxide) donor and NOR-1((±)-(*E*)-methyl-2-[(*E*)-hydroxyimino]-5-nitro-6-methoxy-3-hexenamide).

Smilaside D (**40**), smilaside E (**47**) and smilaside F (**99**) displayed cytotoxicity against human colon tumor (DLD-1) cells (ED_50_ = 2.7, 4.5, 5.0 μg/mL), and smilaside A (**79**) showed weak cytotoxicity against DLD-1 cells (ED_50_ = 11.6 μg/mL). Furthermore, smilaside A (**79**), smilaside B (**107**), smilaside D, smilaside E and smilaside F displayed weak cytotoxicity (ED_50_ = 5.1–13.0 μg/mL) on three to six human tumor cell lines, consisting of human cervical carcinoma (Hela), human oral epithelium carcinoma (KB), DLD-1, human medulloblastoma (Med) cells, human lung carcinoma (A-549) and human breast adenocarcinoma (MCF-7) [14].

### 3.3. Antioxidant Activity

Plenty of CASEDs were found to possess antioxidant activities, mainly related to their substituted acid groups. The antioxidant properties of these compounds were tested by 2,2-diphenyl-1-picrylhydrazyl (DPPH) radical scavenging assays. Probably thanks to the presence of the 3,4-dihydroxy (catechol) moiety in the structure, compound **2** showed significant antioxidant activities, compared to caffeic acid [21]. Compound **21** from *Globularia orientalis* also exhibited antioxidant potential, indicating that it could efficiently scavenge free radicals [32].

Zhang et al. [15] found that smilasides G–L (**38**, **106**, **46**, **41**, **105**, **42**) showed moderate scavenging activities against DPPH radicals and smilasides J–L (**41**, **105**, **42**) exhibited stronger antioxidant activity, which was quite similar to that in positive control ((±)-α-tocopherol). These results support the idea that the substituted feruloyl group plays a key role in the antioxidant activity of phenylpropanoid sugar esters. Heterosmilaside (**95**), helonioside B (**45**) and compound **98** showed strong antioxidant DPPH radical scavenging activity with IC_50_ values of 12.7, 9.1 and 8.7 µg/mL, respectively [46]. Compounds **28**, **32** and **44** exhibited higher activity on scavenging the DPPH radical, compared to l-cysteine at the concentration of 0.02 mM, and the antioxidant activity of compound **32** was almost as same as that of α-tocopherol [36]. Compound **62** and verbascoside showed antioxidant potential pointing out their ability to efficiently scavenge free radicals. 6-*O*-Sinapoyl sucrose (**75**) showed weak activity in the DPPH test, but in the superoxide scavenging test, its antioxidative activity increased slightly, hence, a sucrose moiety esterified by sinapic acid seems to regulate the antioxidative activity [115]. Lapathoside D (**31**) showed DPPH radical scavenging activity with an IC_50_ of 0.088 mM [3]. Kiem et al. [53] found that vanicoside A (**102**), hydropiperoside B (**103**) and vanicoside E (**113**) exhibited significant DPPH radical scavenging properties, with IC_50_ values of 23.4, 26.7 and 49.0 µg/mL, respectively. However, compounds **66**, **67** and **113** were inactive, probably due to the non-existence of acetyl groups in their molecules compared with **102**, **103** and **113**. Wang et al. discovered that diboside A (**58**) and lapathoside A (**63**) only showed low activities in the DPPH test [51]. 

Ehrenoside (**183**), verpectoside A (**185**), B (**193**) and C (**194**) were isolated from the aerial parts of *Veronica pectinata* var. *glandulosa*. They revealed potent radical scavenging activity against DPPH radical. Ehrenoside and verpectoside B were more active than 3-*tert*-butyl-4-hydroxyanisole (BHA) and had comparable activity to all dl-α-tocopherol [104]. Hamerski et al. reported that the antioxidant activity of compound **2** (IC_50_ values 15.0 μM) was comparable to that of the positive control caffeic acid, while compound **253** possess only weak activity [21]. 

In the study of Wang et al. [116], compound **59** possessed modest activity, with an IC_50_ of 20.1 µM in the DPPH radical scavenging test and in the metmyoglobin assay it had antioxidative activity comparable with Trolox (3.70 Trolox equivalents). Quiquesetinerviusides A-E (**86**, **87**, **115**, **85** and **114**) exhibited low DPPH scavenging activity, but considerable·OH radical scavenging activity (IC_50_ 8.4 ± 1.1, 6.8 ± 1.0, 7.4 ± 1.0, 5.5 ± 0.9, 3.6 ± 0.8 µM, respectively) [4]. Hosoya et al. [89] used ESR to evaluate the effect on superoxide anion radicals (O^2−^) of compounds **154**, **150**, **155**, **153** and they exhibited IC_50_ values of 28.5, 84.5, 8.4, 17.1 µM, respectively, using ascorbic acid (IC_50_ value 140 µM) as a positive control. 

### 3.4. Antiinflammatory Activity

Antiinflammatory activity referes to the removal of inflammation or swelling. Acteoside (**131**), angoroside A (**196**) and angoroside C (**200**) revealed a considerable effect in the TXB2-release assay. Angoroside A (**196**), angoroside D (**199**), acteoside (**131**) and isoacteoside (**128**) significantly inhibited LPS-induced PGE2, NO and TNF-α in a concentration-dependent manner. In LPS-stimulated macrophages, angoroside C (**200**) only had activity on NO [63,70]. Acteoside (**131**) had strong in vitro and in vivo anti-inflammatory effects, whilst isoacteoside (**128**) was found to have modest activity. Pretreatment with 1–50 μM CASED (compounds **131**, **157**, **220**) concentration-dependently diminished phorbol-12-myristate-13-acetate (PMA) and *N*-formyl-methionyl-leucyl-phenylalanine (fMLP)-induced reactive oxygen species (ROS) production with IC_50_ values of approximately 6.8–23.9 and 3.0–8.8 μM, respectively [117]. The anti-inflammatory activities of quiquesetinerviusides D (**85**) and E (**114**) were evaluated in RAW 264.7 cells. Both of them exhibited strong activities against LPS-stimulated NO production. And the outcome showed inhibition of quiquesetinerviuside D and E (IC_50_ 9.5, 9.2 µM) compared with a positive control, quercetin (IC_50_ 34.5 µM). In vitro cyclooxygenase (COX) catalyzed prostaglandin biosynthesis inhibition assay, compounds **131**,**205**, **218** and these compounds exhibited stronger inhibitory potencies on Cox-2 than Cox-1 (**131**,**205**, **218** IC_50_ on Cox-2 at 0.69, 0.49 and 0.61 mM, respectively).

### 3.5. Antiviral Activity

Niruriside (**109**) has particular inhibitory activity with an IC_50_ value of 3.3 μM, against the binding of regulation of virion expression (REV) protein to responsive element (RRE) RNA [60]. Kernan et al. [69] reported that verbacteoside (**131**), isoverbascoside (**128**), luteoside A (**188**) and luteoside B (**189**) exhibited antiviral activity (EC_50_) in an in vitro assay against respiratory syncytial virus (RSV), which was resembled or better than that of ribavirin, a drug used to cure RSV contagion in humans. Furthermore, these compounds also showed better activity against RSV than ribavirin. Verbascoside (**131**) exhibited antiviral activity against vesivular stomatitis virus (VSV), but was inactive against herpes simplex type I (HSV-1). The non-toxic confining cellular viability concentration for the activity was 53.6% at 500 μg/mL [118].

### 3.6. Other Activities

Compounds **138**,**131**, **159**, **158** isolated from *Paulownia tomentosa* stems were texted for in vitro cytotoxity against *Streptococcus pyogenes* (A308 and A77), *Staphylococcus aureus* (SG511, 285 and 503), *Streptococcus faecium* MD8b, etc. All the compounds exhibited remarkable antibacterial activity. Compound **159** showed a minimal inhibitory concentration (MIC) value of 150 μg/mL against *Staphylococcus* and *Streptococcus* species [76]. A mixture of poliumoside (**216**) and lamalboside (**227**) revealed moderate antibacterial activity. Compounds **130**, **205** and **218** also possess antimicrobial activity [119]. Vanicoside A (**102**) and B (**67**) showed β-glucosidase inhibitory activity, with IC_50_ values of 59.8 and 48.3 μg/mL (59.9 and 50.5 μM), respectively [120]. The activity of forsythoside B (**205**) and alyssonoside (**206**) against free radical-induced impairment of endothelium-dependent relaxation in isolated rat aorta was investigated. Both provided partial protection at 10^−4^ M concentration against the electrolysis-induced inhibition acetylcholine response [121]. Senegin II (**319**) was tested for hypoglycemic activity in normal and KK-Ay mice. Under similar conditions, senegin II not only reduced the level of blood glucose in normal mice 4 h after intraperitoneal administration, but also significantly lowered the blood glucose level of KK-Ay mice [122]. Tenuifolioses B (**288**), and C (**284**) potentiated basal synaptic transmission in the dentate gyrus of anesthetized rats [12]. The only septsaccharide ester, polygalasaponin XXXII (**334**), could improve hippocampus-dependent learning and memory. The result suggests that it may be through the enhancement of synaptic transmission, activation of the MAP kinase cascade and improvement of BDNF level [56].

The rhizome extracts of *Smilax glabra* Rox B., which is called tufuling in Traditional Chinese Medicine, show many kinds of pharmacological activities like hypoglyceaemic, immuno- modulatory, free-radical scavenging and antioxidant enzyme fortifying activities. Compounds **32**, **90**, **100**, **101**, **108**, **111**, **112** were purified from the *S*. *glabra* which should impulse scientistc to perform more research on these compounds [40].

## 4. Conclusions 

Because of the wide range of distribution, diverse structures and significant pharmacological activities of the CASEDs, more natural product researchers are paying great attention to these compounds. However, most studies on the CASED since 1977 are still isolated and report simple pharmacological activities. More in-depth research on the pharmacological mechanisms of action should be performed. Full exploitation on the broad array of biological activities of CASEDs awaits more researchers to devote themselves to this field.

## Figures and Tables

**Table:** 

Cpd.	R_1_	R_2_	R_3_	R_4_	R_5_	Cpd.	R_1_	R_2_	R_3_	R_4_	R_5_
**1**	E	H	H	H	G	**6**	R	H	H	E	H
**2**	G	H	H	H	G	**7**	S	H	H	H	G
**3**	S	H	H	H	I	**8**	S	H	H	G	H
**4**	I	H	H	H	I	**9**	T	H	H	H	G
**5**	R	H	H	H	E	**10**	V	H	H	H	G

**Table:** 

Cpd.	R1	R2	R3	R4
**11**	H	H	H	F
**12**	H	A	F	H
**13**	H	A	F	F
**14**	H	F	A	H
**15**	H	A	F′	H

**Table:** 

Cpd.	R_1_	R_2_	R_3_	R_4_	R_5_
**16**	H	H	H	H	E
**17**	H	H	H	H	G
**18**	H	H	H	H	K
**19**	H	H	H	E	H
**20**	H	H	G′	H	G

**Table:** 

Cpd.	R_1_	R_2_	R_3_	R_4_	R_5_	R_6_	R_7_	R_8_	Cpd.	R_1_	R_2_	R_3_	R_4_	R_5_	R_6_	R_7_	R_8_
**28**	H	H	H	H	H	H	H	I	**75**	H	H	H	K	H	H	H	H
**29**	H	H	H	H	H	H	H	K	**76**	H	H	H	K	H	H	H	I
**30.**	H	H	H	H	H	H	H	M	**77**	H	H	H	M	H	H	H	M
**31**	H	H	H	H	H	E	H	E	**78**	H	H	H	M	H	H	H	H
**32**	H	H	H	H	H	I	H	I	**79**	H	H	A	A	H	I	H	I
**33**	H	H	H	H	H	I	H	E	**80**	H	H	A	B	H	H	H	M
**34**	H	H	H	H	K	H	H	I	**81**	H	H	A	I	H	H	H	I
**35**	H	H	H	H	K	H	H	K	**82**	H	H	A	K	H	H	H	K
**36**	H	H	H	H	A	H	H	I	**83**	H	H	B	H	H	H	H	I
**37**	H	H	H	H	E	E	H	E	**84**	H	H	B	H	H	H	H	M
**38**	H	H	H	H	E	I	H	E	**85**	H	H	E	A	H	I	H	I
**39**	H	H	H	H	E	I	H	I	**86**	H	H	I	H	H	I	H	I
**40**	H	H	H	H	E	I	A	I	**87**	H	H	I	A	H	I	H	I
**41**	H	H	H	H	I	I	H	E	**88**	H	H	K	H	H	H	H	K
**42**	H	H	H	H	I	I	H	I	**89**	H	A	H	A	A	H	H	E
**43**	H	H	H	A	H	H	H	M	**90**	H	A	H	A	H	I	H	I
**44**	H	H	H	A	H	H	H	I	**91**	H	A	H	I	E	E	H	E
**45**	H	H	H	A	H	I	H	I	**92**	H	A	H	I	H	A	H	I
**46**	H	H	H	A	E	I	H	E	**93**	H	A	H	I	H	A	A	I
**47**	H	H	H	A	E	I	H	I	**94**	H	A	H	K	H	H	H	K
**48**	H	H	H	B	H	H	H	I	**95**	H	I	H	H	H	I	H	H
**49**	H	H	H	B	H	H	H	M	**96**	H	A	A	A	A	H	H	E
**50**	H	H	H	B	H	H	H	K	**97**	A	H	H	A	A	I	H	I
**51**	H	H	H	C	H	H	H	M	**98**	A	H	H	A	H	I	H	I
**52**	H	H	H	C	H	H	H	K	**99**	A	H	H	A	I	I	H	E
**53**	H	H	H	D	H	H	H	H	**100**	A	H	H	A	I	I	H	I
**54**	H	H	H	E	H	H	H	K	**101**	A	H	H	A	E	I	H	I
**55**	H	H	H	E	H	H	H	M	**102**	A	H	H	I	E	E	H	E
**56**	H	H	H	E	H	H	I	I	**103**	A	H	H	I	I	E	H	E′
**57**	H	H	H	E	E	E	H	E	**104**	A	H	H	H	E	E	H	E
**58**	H	H	H	E	E	I	H	E	**105**	A	H	H	H	E	I	H	I
**59**	H	H	H	G	H	H	H	H	**106**	A	H	H	H	E	I	H	E
**60**	H	H	H	G	H	H	I	I	**107**	A	H	H	H	H	I	H	I
**61**	H	H	H	I	H	H	H	M	**108**	A	H	A	A	H	I	H	I
**62**	H	H	H	I	H	H	H	H	**109**	A	H	A	A	A	D	H	D
**63**	H	H	H	I	I	E	H	E	**110**	A	H	A	A	A	I	H	I
**64**	H	H	H	I	I	I	H	E	**111**	A	H	A	A	I	I	H	I
**65**	H	H	H	I	H	E	H	E	**112**	A	H	A	A	E	I	H	I
**66**	H	H	H	I	E	E	H	H	**113**	A	H	A	I	E	E	H	E
**67**	H	H	H	I	E	E	H	E	**114**	A	H	E	H	H	I	H	I
**68**	H	H	H	I	H	H	A	I	**115**	A	H	I	H	H	I	H	I
**69**	H	H	H	I	H	A	H	I	**116**	A	A	A	A	H	H	H	E
**70**	H	H	H	I	H	A	A	I	**117**	A	A	H	A	A	H	H	E
**71**	H	H	H	I	H	H	H	I	**118**	E	H	A	H	A	E	H	E
**72**	H	H	H	K	H	H	H	M	**119**	I	H	H	H	H	I	H	I
**73**	H	H	H	K	H	H	H	K	**120**	A	A	A	A	H	H	H	G
**74**	H	H	H	K	H	H	K	K									

**Table:** 

Cpd.	R_1_	R_2_	R_3_	R_4_
**121**	S	G	H	H

**Table:** 

Cpd.	R_1_	R_2_	R_3_	R_4_	R_5_	R_6_	R_7_	Cpd.	R_1_	R_2_	R_3_	R_4_	R_5_	R_6_	R_7_
**122**	R	H	H	E	H	H	H	**132**	S	H	G′	H	H	H	H
**123**	R	H	E	H	H	H	H	**133**	S	H	I	H	H	H	H
**124**	G	H	T	H	H	H	H	**134**	S	H	I′	H	H	H	H
**125**	Q	H	G	H	H	H	H	**135**	S	A	G	H	H	H	A
**126**	R	H	E	H	H	H	H	**136**	S	A	G	H	H	H	H
**127**	S	H	H	E	H	H	H	**137**	T	H	A	I	H	A	H
**128**	S	H	H	G	H	H	H	**138**	T	H	I	H	H	H	H
**129**	S	H	E′	H	H	H	H	**139**	T	H	I′	H	H	H	H
**130**	S	H	G	D	H	H	H	**140**	U	H	I	H	H	H	H
**131**	S	H	G	H	H	H	H								

**Table:** 

Cpd.	R_1_	R_2_	R_3_	R_4_
**141**	S	H	G	H

**Table:** 

Cpd.	R_1_	R_2_	R_3_	R_4_
**142**	S	H	H	G
**143**	S	H	G	H

**Table:** 

Cpd.	R_1_	R_2_	R_3_	R_4_
**144**	S	H	G	H

**Table:** 

Cpd.	R_1_	R_2_	R_3_	R_4_
**145**	H	H	H	E
**146**	H	H	G	H
**147**	T	H	H	I
**148**	S	H	H	G

**Table:** 

Cpd.	R_1_	R_2_	R_3_	R_4_
**148**	S	H	H	G

**Table:** 

Cpd.	R_1_	R_2_	R_3_
**149**	D	H	R

**Table:** 

Cpd.	R_1_	R_2_
**150**	G	K
**151**	I	K
**152**	K	K
**153**	L	I
**154**	L	K
**155**	L	L

**Table:** 

Cpd.	R_1_	R_2_	R_3_	Cpd.	R_1_	R_2_	R_3_
**163**	K	H	K	**165**	M	I	K
**164**	M	H	K	**166**	M	K	K

**Table:** 

Cpd.	R_1_	R_2_	R_3_	R_4_	R_5_	R_6_	R_7_
**167**	H	H	I	H	H	H	K
**168**	H	H	K	H	H	H	K

**Table:** 

Cpd.	R_1_	R_2_	Cpd.	R_1_	R_2_
**169**	I	H	**171**	H	I
**170**	K	H	**172**	H	K

**Table:** 

Cpd.	R_1_	R_2_	R_3_	R_4_	R_5_	R_6_	R_7_	R_8_
**173**	H	H	H	O	H	H	A	I
**174**	H	H	P	B	H	H	H	B

**Table:** 

Cpd.	R_1_	R_2_	R_3_
**175**	E	A	D

**Table:** 

Cpd.	R_1_	R_2_	R_3_	R_4_
**176**	S	H	H	G
**177**	S	H	G	H

**Table:** 

Cpd.	R_1_	R_2_
**178**	S	G

**Table:** 

Cpd.	R_1_	R_2_
**179**	S	G
**180**	S	I
**181**	T	I
**182**	U	I

**Table:** 

Cpd.	R_1_	R_2_	R_3_
**183**	S	H	G
**184**	S	G	H
**185**	S	G	A
**186**	S	I	H
**187**	T	I	H

**Table:** 

Cpd.	R_1_	R_2_	R_3_
**188**	S	G	H
**189**	S	G	A
**190**	S	H	G
**191**	S	H	I
**192**	T	H	I

**Table:** 

Cpd.	R_1_	R_2_	R_3_
**193**	S	G	A

**Table:** 

Cpd.	R_1_	R_2_	R_3_
**194**	S	G	H
**195**	S	I	H

**Table:** 

Cpd.	R_1_	R_2_	R_3_
**196**	U	H	I

**Table:** 

Cpd.	R_1_	R_2_	R_3_
**197**	S	H	G
**198**	S	H	I
**199**	S	H	I′
**200**	S	H	I
**201**	T	H	I

**Table:** 

Cpd.	R_1_	R_2_	R_3_
**202**	S	H	G
**203**	U	H	G
**204**	U	H	I
**205**	U	H	I′

**Table:** 

Cpd.	R_1_	R_2_	R_3_
**206**	S	H	G
**207**	S	H	I

**Table:** 

Cpd.	R_1_	R_2_	R_3_
**208**	S	H	E
**209**	S	H	G
**210**	S	H	I
**211**	S	H	I′
**212**	U	H	I
**213**	U	H	I′

**Table:** 

Cpd.	R_1_	R_2_	R_3_
**214**	S	H	G
**215**	U	H	G
**216**	U	H	I

**Table:** 

Cpd.	R_1_	R_2_	R_3_
**217**	S	H	G
**218**	S	A	G

**Table:** 

Cpd.	R_1_	R_2_	R_3_
**219**	S	H	G

**Table:** 

Cpd.	R1	R2	R3	R4
**220**	S	H	G	H

**Table:** 

Cpd.	R1	R2	R3	R4
**221**	E	H	I	H
**222**	S	H	G	H
**223**	S	A	G	H

**Table:** 

Cpd.	R1	R2	R3	R4
**224**	H	H	I	H

**Table:** 

Cpd.	R1	R2	R3	R4
**225**	S	H	G	H
**226**	S	H	I	H
**227**	T	H	I	H

**Table:** 

Cpd.	R1	R2	R3	R4
**228**	S	H	G	H

**Table:** 

Cpd.	R1	R2	R3	R4
**229**	S	H	G	H

**Table:** 

Cpd.	R1	R2	R3	R4
**230**	S	H	G	H

**Table:** 

Cpd.	R1	R2	R3	R4
**231**	S	H	G	H
**232**	S	H	I	H

**Table:** 

Cpd.	R_1_	R_2_	R_3_	R_4_
**233**	R	H	H	E
**234**	R	H	H	G

**Table:** 

Cpd.	R_1_	R_2_	R_3_	R_4_
**235**	H	H	G	H
**236**	R	H	E	H
**237**	R	H	G	H
**238**	S	H	G	H

**Table:** 

Cpd.	R_1_	R_2_	R_3_	R_4_
**239**	S	H	G	H
**240**	S	H	I	H

**Table:** 

Cpd.	R_1_	R_2_	R_3_	R_4_
**241**	S	H	G	H
**242**	T	H	I	H

**Table:** 

Cpd.	R_1_	R_2_	R_3_
**243**	S	G	H

**Table:** 

Cpd.	R_1_	R_2_	R_3_
**244**	S	G	H

**Table:** 

Cpd.	R_1_	R_2_	R_3_
**245**	T	H	I

**Table:** 

Cpd.	R_1_	R_2_	R_3_
**246**	S	G	H

**Table:** 

Cpd.	R_1_	R_2_	Cpd.	R_1_	R_2_
**249**	D	R	**252**	F	R
**250**	E	R	**253**	I	R
**251**	E′	R	**254**	J	R

**Table:** 

Cpd.	R_1_	R_2_	R_3_	R_4_	Cpd.	R_1_	R_2_	R_3_	R_3_
**255**	K	H	H	K	**258**	K	K	H	K
**256**	K	E	H	K	**259**	M	I	H	K
**257**	K	I	H	K	**260**	M	K	H	K

**Table:** 

Cpd.	R_1_	R_2_	R_3_	R_4_	R_5_	R_6_
**261**	I	B	H	I	A	A

**Table:** 

Cpd.	R_1_	R_2_	R_3_	R_4_	R_5_	Cpd.	R_1_	R_2_	R_3_	R_4_	R_5_
**262**	E	B	I	A	A	**269**	E	B	I	H	I
**263**	E	B	H	E	A	**270**	E	B	I	A	I
**264**	E	B	E	H	A	**271**	I	B	E	A	A
**265**	E	B	E	A	A	**272**	I	B	I	H	A
**266**	E	B	E	A	E	**273**	I	B	I	A	H
**267**	E	B	I	H	A	**274**	I	B	I	A	A
**268**	E	B	I	H	E						

**Table:** 

Cpd.	R_1_	R_2_	R_3_
**275**	J	H	H

**Table:** 

Cpd.	R_1_	R_2_	R_3_
**276**	S	G	H

**Table:** 

Cpd.	R_1_	R_2_	R_3_	R_4_	R_5_	R_6_	Cpd.	R_1_	R_2_	R_3_	R_4_	R_5_	R_6_
**280**	E	B	E	H	H	A	**288**	E	B	I	A	A	A
**281**	E	B	E	H	A	A	**289**	I	B	I	H	H	A
**282**	E	B	E	A	H	A	**290**	I	B	I	H	A	A
**283**	E	B	E	A	A	A	**291**	I	B	I	A	H	A
**284**	E	B	I	H	H	A	**292**	I	B	I	A	A	A
**285**	E	B	I	H	A	A	**293**	I	B	E′	A	H	A
**286**	E	B	I	A	H	A	**294**	I	B	H	I	H	A
**287**	E	B	I	A	H	H							

**Table:** 

Cpd.	R_1_	R_2_	R_3_	R_4_	R_5_	Cpd.	R_1_	R_2_	R_3_	R_4_	R_5_
**295**	E	B	I	A	H	**301**	I	B	I	H	H
**296**	E	B	I	A	A	**302**	I	B	I	H	A
**297**	E	B	I′	A	A	**303**	I	B	I	A	H
**298**	E′	B	I	A	A	**304**	I	B	I	A	A
**299**	I	B	E	A	H	**305**	I	B	I′	A	A
**300**	I	B	E	A	A						

**Table:** 

Cpd.	R_1_	R_2_	R_3_	R_4_	R_5_
**306**	E	B	N	A	H
**307**	E	B	N	A	A
**308**	E	B	P	A	H
**309**	E	B	P	A	E
**310**	E	B	P	A	I
**311**	I	B	N	A	H
**312**	I	B	P	H	I
**313**	I	B	P	A	H
**314**	I	B	P	A	A
**315**	I	B	P	A	E
**316**	I	B	P	A	I

**Table:** 

Cpd.	R_1_	R_2_	R_3_
**317**	F	H	H
**318**	F′	H	H
**319**	J	H	H
**320**	J′	H	H

**Table:** 

Cpd.	R_1_	R_2_	R_3_	R_4_	R_5_
**321**	E	B	A	H	A
**322**	E	B	A	A	A

**Table:** 

Cpd.	R_1_	R_2_	R_3_	R_4_	R_5_
**323**	E	B	A	H	A
**324**	E	B	A	A	A

**Table:** 

Cpd.	R_1_	R_2_	R_3_	R_4_
**325**	J	H	H	H
**326**	J	A	H	H
**327**	J′	A	H	H
**328**	F	H	H	H

**Table:** 

Cpd.	R_1_	R_2_	R_3_	R_4_
**329**	F	H	H	H

**Table:** 

Cpd.	R_1_	R_2_	R_3_
**330**	M	H	H

**Table:** 

Cpd.	R_1_	R_2_
**331**	F	H
**332**	F′	H

**Table:** 

Cpd.	R_1_	R_2_	R_3_	R_4_	R_5_
**333**	E	B	A	A	A

**Table:** 

Cpd.	R_1_	R_2_
**334**	F	H

**Table 1:** The Family Distribution of CASEDs.

Family	Number	Family	Number
*Asclepiadaceae*	1	*Gesneriaceae*	8
*Hymenophyllaceae*	1	*Labiatae*	11
*Sterculiaceae*	1	*Lamiaceae*	11
*Amaranthaceae*	2	*Rosaceae*	13
*Smilacaeae*	3	*Orobanchaceae*	16
*Magnoliaceae*	3	*Polygonaceae*	19
*Rubiaceae*	5	*Oleaceae*	20
*Plantaginaceae*	6	*Bignoniaceae*	22
*Cruciferae*	6	*Liliaceae*	34
*Verbenaceae*	7	*Scrophulariaccae*	58
*Polygalaceae*	126		

**Table 2:** The Principal Compounds of CASEDs Distributed in TCMs.

Name in TCM	Sources	Traditional Effect	Medicinal Parts	Compounds	Activity	Refs.
Polygalae Radix	*Polygala tenuifolia* Willd.	Common wisdom calms the nerves, restoring normal coordination between heart and kidney, Expectoration, subsidence of a swelling	Root	**51, 52, 72, 73, 280–290, 292, 321–324**	Anti-depression activity, neuroprotective activity	[10,11,12]
	*Polygala sibirica* L.			**28–30, 50, 51, 73, 75, 78, 88**	Anti-depression activity, neuroprotective activity, antioxidant activity	[13]
Smilacis China Rhizoma	*Smilaz china* L.	Syphilis, gout, and rheumatism	Root	**39, 40, 45, 47, 79, 98, 99, 101, 107**	Anticancer activity	[14]
	*Smilax bracteata* C. Presl			**38, 41, 42, 45–47, 105, 106**	Antioxidant activity	[15]
Scrophula-riae Radix	*Scrophularia ningpoensis* Hemsl.	Clearing heat and cooling blood, nourishing yin to reduce pathogenic fire, detoxicating and resolving a mass	Root	**14, 53, 59, 132**	Antioxidative activity	[16,17]
Scrophula-riae Radix	*Scrophularia buergeriana* Miq.	Clearing heat and cooling blood, nourishing yin to reduce pathogenic fire, detoxicating and resolving a mass	Root	**11, 12, 13, 15**	Neuroprotective	[18]
Rehmann-ia Radix	*Rehmannia glutinosa* var. *Purpurea*	Clearing heat and cooling blood, promoting the secretion of saliva or body fluid	Root	**124, 125, 131, 133, 136, 138, 207–212**	PKC inhibitory activity, antiinflammatory effects, antiviral activity, antibacterial activity	[19]

**Table 3:** Cinnamic Acid Sugar Ester Derivatives.

No.	Name	Source	Refs.
**1**	6-*O*-Caffeoyl-1-*O*-*p*-coumaroyl-β-d-glucopyranose	*Prunus buergeriana*	[20]
**2**	1,6-Di-*O*-caffeoyl-β-d-glucopyranose	*Prunus buergeriana*; *Coussarea hydrangeifolia*	[20,21]
**3**	Osmanthuside E	*Osmanthus asiaticus*	[22]
**4**	1,6-Diferuloyl glucose	*Sterculia foetida*	[23]
**5**	Eutigoside A	*Ligustrum purpurascens*	[24]
**6**	Osmanthuside A	*Ligustrum purpurascens*	[24]
**7**	2-(3,4-Dihydroxyphenyl)-ethyl-(6-*O*-caffeoyl)-β-d-glucopyranoside or calceolarioside B	*Calceolaria hypericina*; *Prunus ssiori*; *Paraboea glutinosa*	[25,26]
**8**	3,4-Dihydroxyphenethyl alcohol 4-*O*-Caffeoyl-β-d-allopyranoside or calceolarioside A or derhamnosylverbascoside	*Trichomanes reniforme* Forst.f; *Calceolaria hypericina*; *Lantana camaro* L.	[25,27,28]
**9**	1′-*O*-β-d-(1-Hydroxy-4-oxo-2,5-cyclohexadien)-ethyl-6′-*O*-caffeoylglucopyranoside or calceolarioside D	*Calceolaria hypericina*	[25]
**10**	2-(3,4-Methylenedioxyphenyl)-ethyl-(6-*O*-caffeoyl)-β-d-glucopyranoside	*Prunus ssiori*	[29]
**11**	4-*O*-(*E*)-*p*-Methoxycinnamoyl-α-l-rhamno-pyranoside or buergeriside C_3_	*Scrophularia buergeriana*	[18]
**12**	2-*O*-Acetyl-3-*O*-(*E*)-*p*-methoxycinnamoyl-α-l-rhamnopyranoside or buergeriside B_1_	*Scrophularia buergeriana*	[18]
**13**	2-*O*-Acetyl-3,4-di-*O*-(*E*)-*p*-methoxycinnamoyl-α-l-rhamnopyranoside or buergeriside A_1_	*Scrophularia buergeriana*	[18]
**14**	3-*O*-Acetyl-2-*O*-*p*-methoxycinnamoyl-α(β)-l-rhamnopyranose or ningposide D	*Scrophularia ningpoensis*	[16]
**15**	2-*O*-Acetyl-3-*O*-(*Z*)-*p*-methoxycinnamoyl-α-l-rhamnopyranoside or buergeriside B_2_	*Scrophularia buergeriana*	[18]
**16**	6-*O*-*p*-Coumaroyl-d-glucopyranose	*Prunus buergeriana*	[20]
**17**	6-*O*-Caffeoyl-d-glucopyranose or 6-*O*-Caffeoyl-d-glucopyranoside	*Prunus buergeriana*; *Prunus ssiori*	[20,29]
**18**	6-*O*-[*E*]-Sinapoyl-(α- and β-)-d-glucopyranoside	*Cynanchum hancockianum*	[30]
**19**	*O*-Acylglycoses	*Ligustrum purpurascens*	[24]
**20**	3,6-di-*O*-Caffeoyl-(α/β)-glucose	*Rubus sanctus*	[31]
**21**	6-*O*-Feruloyl-β-d-glucopyranosyl-(1→6)-glucitol or globularitol	*Globularia orientalis*	[32]
**22**	(2*R*)-[(6-*O*-Caffeoyl)-β-d-glucopyranosyloxy]-benzeneacetonitrile or grayanin	*Prunus buergeriana*	[20]
**23**	Scrophyloside A	*Neopicrorhiza scrophulariiflora*	[33]
**24**	Scrophyloside B	*Neopicrorhiza scrophulariiflora*	[33]
**25**	Hexane-1,2,3,4,5-pentanol 1-*O*-β-(6-*O*-(*E*)-feruloyl) glucopyranoside or paederol A	*Paederia scandens*	[34]
**26**	Butane-1,2,3,4-tetraol 1-*O*-β-(6-*O*-(*E*)-feruloyl) glucopyranoside or paederol B	*Paederia scandens*	[34]
**27**	Kaempferol 3-*O*-β-d-(6-*O*-*p*-*E*-Coumaroyl)-glucopyranoside	*Froelichia floridana*	[35]
**28**	3-*O*-Feruloylsucrose or sibiricose A_5_	*Trillium kamtschaticum*; *Polygala sibirica*	[13,36]
**29**	3′-Sinapoyl sucrose or sibiricose A_6_	*Polygala sibirica*; *Polygala tricornis*	[13,37]
**30**	3-*O*-[(*E*)-3,4,5-Trimethoxycinnamoyl]-β-d-fructo-furanosyl-(2→1)-α-d-glucopyranoside or glomeratose A	*Polygala sibirica*; *Polygala tricornis*; *Polygala glomerata*	[13,37,38]
**31**	3,6-Di-*p*-coumaroyl sucrose or lapathosides D	*Polygonum lapathifolium*	[39]
**32**	Heronioside A	*Trillium kamtschaticum*; *Smilax glabra*	[36,40]
**33**	Parispolyside F	*Paris polyphylla* var. *yunnanensis*	[41]
**34**	β-d-(1-Sinapoyl-3-feruloyl)-α-d-glucopyranoside	*Polygala chamaebuxus*	[42]
**35**	β-d-(l-Acetyl-3-feruloyl)-fructofuranosyl-α-d-gluco-pyranoside	*Polygala chamaebuxus*	[42]
**36**	β-d-(1,3-Disinapoyl)-fructofuranosyl-d-gluco-pyranoside	*Polygala chamaebuxus*	[42]
**37**	β-d-(1,3,6-Tri-p-coumaryl)-fructofuranosyl-α-d-glucopyranoside or hydropiperoside	*Polygonum hydropiperitum*; *Polygonurn hydropiper*	[39,43]
**38**	(1,3-*O*-di-*p*-Coumaroyl-6-*O*-feruloyl)-β-d-fructo-furanosyl-(2→1)-α-d-glucopyranoside or smilaside G	*Smilax bracteata*	[15]
**39**	1-*p*-Coumaroyl-3,6-diferuloyl sucrose or smilaside C	*Smilax china*	[14]
**40**	1-*p*-Coumaroyl-3,6-diferuloyl-4-acetyl sucrose or smilaside D	*Smilax china*	[14]
**41**	(3-*O*-*p*-Coumaroyl-1,6-*O*-diferuloyl)-β-d-fructo-furanosyl-(2→1)-α-d-glucopyranoside or smilaside J	*Smilax bracteata*	[15]
**42**	1,3,6-*O*-Triferuloyl-β-d-fructofuranosyl-(2→1)-α-d-glucopyranoside or smilaside L	*Smilax bracteata*	[15]
**43**	3-*O*-[(*E*)-3,4,5-Trimethoxycinnamoyl]-β-d-fructo-furanosyl-(2→1)-(6-*O*-acetyl)-α-d-glucopyranoside or tricornose A	*Polygala tricornis*	[37]
**44**	Regaloside A	*Trillium kamtschaticum*	[36]
**45**	6′-Acetyl-3,6-diferuloylsucrose or helonioside B	*Smilax china*; *Smilax bracteata*; *Polygonum perfoliatum*; *Heterosmilax erythrantha*	[14,15,44,45]
**46**	(1,3-*O*-di-*p*-Coumaroyl-6-*O*-feruloyl)-β-d-fructo-furanosyl-(2→1)-(6-*O*-acetyl)-α-d-glucopyranoside or smilaside I	*Smilax bracteata*	[15]
**47**	1-*p*-Coumaroyl-3,6-diferuloyl-6′-acetyl sucrose or smilaside E	*Smilax china*; *Smilax bracteata*	[14,15]
**48**	Reiniose C	*Polygala reinii* Fr.*et* Sav	[46]
**49**	6-*O*-Benzoyl-3′-*O*-3,4,5-trimethoxycinnamoyl-sucrose or 3-*O*-[(*E*)-3,4,5-trimethoxy-cinnamoyl]-β-d-fructofuranosyl-(2→1)-(6-*O*-benzoyl)-α-d-glucopyranoside or [3-*O*-(3,4,5-trimethoxycinnamoyl]-β-d-fructo-furanosyl-(6-*O*-benzoyl)-α-d-glucopyranoside	*Polygala tricornis*; *Polygala glomerata*; *Polygala reinii* Fr.*et* Sav	[37,38,46]
**50**	3′-Sinapoyl-6-benzoyl sucrose or 6-*O*-benzoyl-3′-*O*-sinapoylsucrose 6-*O*-benzoyl-3′-*O*-sinapoylsucrose or (3-*O*-[(2*E*)-3-(4-hydroxy-3,5-dimethoxyphenyl)-1-oxoprop-2-enyl]-β-d-fructofuranosyl 6-*O*-benzoyl-α-d-glucopyranoside)	*Polygala sibirica*; *Polygala tricornis*; *Polygala telephioides*Willd.	[13,37,47]
**51**	β-d-[3-*O*-(3,4,5-Trimethoxycinnamoyl)]-fructo-furanosyl-α-D-[6-*O*-(*p*-hydroxybenzoyl)]-gluco-pyranoside or tenuifoliside A	*Polygala tenuifolia*; *Polygala sibirica*	[10,11,12,13]
**52**	β-d-(3-*O*-Sinapoyl)-fructofuranosyl-α-d-(6-*O*-(*p*-hydroxybenzoyl)]-glucopyranoside or tenuifoliside B	*Polygala tenuifolia*	[10]
**53**	Sibirioside A	*Scrophularia ningpoensis* Hemsl	[17]
**54**	3-*O*-(*E*)-Sinapoyl-β-d-fructofuranosyl-(2→1)-[6-*O*-(*E*)-*p*-coumaroyl]-α-d-glucopyranoside or glomeratose B	*Polygala glomerata*	[38]
**55**	3-*O*-[(*E*)-3,4,5-Trimethoxycinnamoyl]-β-d-fructo-furanosyl-(2→1)-[6-*O*-(*E*)-*p*- coumaroyl] -α-d-glucopyranoside or glomeratose C	*Polygala glomerata*	[38]
**56**	3,4-*O*-β-d-Di-feruloyl-fructofuranosyl-6-*O*-α-d-(*p*-coumaroyl)-glucopyranoside	*Monnina obtusifolia* H.B.K.	[48]
**57**	6′-*O*-*p*-Coumarylhydropiperoside or vanicoside D	*Polygonum pensylvanicum*	[49]
**58**	1,3,6′-Tri-*p*-coumaroyl-6-feruloyl sucrose or diboside A	*Fagopyrum dibotrys* (D. Don.) Hara.	[50]
**59**	6-*O*-Caffeoyl-β-d-fructofuranosyl-(2→1)-α-d-gluco-pyranoside	*Scrophularia ningpoensis* Hemsl; *Globularia orientalis*	[17,32]
**60**	3,4-*O*-β-d-Di-feruloyl-fructofuranosyl-6-*O*-α-d-(caffeoyl)-glucopyranoside	*Monnina obtusifolia* H.B.K.	[48]
**61**	Reiniose A	*Polygala reinii* Fr.*et* Sav	[46]
**62**	6-*O*-Feruloyl-β-d-fructofuranosyl-(2→1)-α-d-glucopyranoside or β-d-fructofuranosyl-6-*O*-feruloyl-α-d-glucopyranoside or arillatose B	*Globularia orientalis*; *Polygala arillata*	[32,51]
**63**	1,6′-Diferuloyl-3,6-di-*p*-coumaroylsucrose or lapathoside A	*Polygonum lapathifolium*	[39]
**64**	1,6,6′-Triferuloyl-3-*p*-coumaroyl sucrose or lapathoside B	*Polygonum lapathifolium*	[39]
**65**	6′-Feruloyl-3,6-di-*p*-coumaroyl sucrose or lapathoside C	*Polygonum lapathifolium*	[39]
**66**	6′-Feruloyl-1,6-di-*p*-coumaroyl sucrose or hydropiperoside A	*Polygonum hydropiper* L.	[52]
**67**	Vanicoside B	*Polygonum perfoliatum*; *Polygonum pensylvanirum*	[44,53]
**68**	4-Acetyl-3,6′-diferuloylsucrose	*Lilium speciosum* var. *rubrum*; *Lilium longiflorum*	[54,55]
**69**	6-Acetyl-3,6′-diferuloylsucrose	*Lilium speciosum* var. *rubrum*	[54]
**70**	4,6-Diacetyl-3,6′-diferuloylsucrose	*Lilium speciosum* var. *rubrum*	[54]
**71**	3,6′-Diferuloylsucrose	*Lilium speciosum* var. *rubrum*;*Lilium longiflorum*	[54,55]
**72**	β-d-[3-*O*-(3,4,5-Trimethoxycinnamoyl)]-fructo-furanosyl-α-d-(6-*O*-sinapoyl)-glucopyranoside or tenuifoliside C	*Polygala tenuifolia*; *Polygala tricornis*; *Polygala glomerata*; *Polygala reinii* Fr.*et* Sav; *Polygala japonica* Houtt.	[10,37,38,46,56]
**73**	3′,6-Disinapoyl sucrose or 3-*O*-(*E*)-sinapoyl-β-d-fructofuranosyl-(2→1)-[6-*O*-(*E*)-sinapoyl]-α-d-glucopyranoside	*Polygala tenuifolia*; *Polygala sibirica*; *Polygala tricornis*; *Polygala glomerata*; *Polygala reinii* Fr.*et* Sav; *Securidaca longipedunculata*; *Polygala virgata*	[10,13,37,38,46,57,58]
**74**	β-D-(3,4-Disinapoyl)fructofuranosyl-α-d-(6-sinapoyl)glucopyranoside	*Securidaca longipedunculata*	[57]
**75**	6-*O*-Sinapoylsucrose or sibiricose A_1_	*Polygala sibirica*	[13]
**76**	3-*O*-Feruloyl-β-d-fructofuranosyl-(6-*O*-sinapoyl)-α-d-glucopyranoside	*Polygala reinii* Fr.*et* Sav	[46]
**77**	3-*O*-[(*E*)-3,4,5-Trimethoxycinnamoyl]-β-d-fructo-furanosyl-(2→1)-[6-*O*-(*E*)-*p*-coumaroyl]-α-d-glucopyranoside or glomeratose D	*Polygala glomerata*	[38]
**78**	6-*O*-3,4,5-Trimethoxycinnamoyl sucrose or sibiricose A_2_	*Polygala sibirica*	[13]
**79**	3,6-Diferuloyl-4′,6′-diacetylsucrose or smilaside A	*Smilax china*	[14]
**80**	3-*O*-[(*E*)-3,4,5-Trimethoxycinnamoyl]-β-d-fructo-furanosyl-(2→1)-(4-*O*-acetyl)-(6-*O*-benzoyl)-α-d-glucopyranoside or tricornoses B	*Polygala tricornis*	[37]
**81**	4′-Acetyl-3,6′-diferuloylsucrose	*Lilium speciosum* var. *rubrum*	[54]
**82**	β-d-(3-*O*-Sinapoyl)fructofuranosyl-α-d-(4-*O*-acetyl-6-*O*-sinapoyl)glucopyranoside	*Polygala virgata*	[58]
**83**	Reiniose B	*Polygala reinii* Fr.*et* Sav	[46]
**84**	4-*O*-Benzoyl-3′-3,4,5-trimethoxycinnamoylsucrose or [3-*O*-(3,4,5-trimethoxycinnamoyl)]-β-d-fructofuranosyl-(4-*O*-benzoyl)-α-d-gluco-pyranoside	*Polygala tricornis*; *Polygala reinii* Fr.*et* Sav	[37,46]
**85**	(3,6-*O*-Diferuloyl)-β-d-fructofuranosyl-(2→1)-(4-*O*-*p*-coumaroyl-6-*O*-acetyl)-α-d-glucopyranoside or quiquesetinerviuside D	*Calamus quiquesetinervius* Burret	[4]
**86**	(3,6-*O*-Diferuloyl)-β-d-fructofuranosyl-(2→1)-(4-*O*-feruloyl)-α-d-glucopyranoside or quiquesetinerviuside A	*Calamus quiquesetinervius* Burret	[4]
**87**	(3,6-*O*-Diferuloyl)-β-d-fructofuranosyl-(2→1)-(4-*O*-feruloyl-6-*O*-acetyl)-α-d-glucopyranoside or quiquesetinerviuside B	*Calamus quiquesetinervius* Burret	[4]
**88**	3′,4-*O*-Disinapoylsucrose or sibiricose A_4_	*Polygala sibirica*	[13]
**89**	1-*O*-Acetyl-3-*O*-*p*-coumaroyl-β-d-fructofuranosyl-3,6-di-*O*-acetyl-α-d-glucopyranoside	*Prunus padus*	[58]
**90**	(3,6-Di-*O*-feruloyl)-β-d-fructofuranosyl-(3,6-di-*O*-acetyl)-α-d-glucopyranoside	*Smilax glabra*	[40]
**91**	3′-*O*-Acetylvanicoside B or vanicoside F	*Polygonum pensylvanicum*	[49]
**92**	6,3′-Diacetyl-3,6′-diferuloylsucrose	*Lilium speciosum* var. *rubrum*	[54]
**93**	4,6,3′-Triacetyl-3,6′-diferuloylsucrose	*Lilium speciosum* var. *rubrum*	[54]
**94**	β-d-(3-*O*-Sinapoyl)fructofuranosyl-α-d-(3-*O*-acetyl-6-*O*-sinapoyl)glucopyranoside	*Polygala virgata*	[59]
**95**	Heterosmilaside	*Heterosmilax erythrantha*	[45]
**96**	1-*O*-Acetyl-3-*O*-*p*-coumaroyl-β-d-fructofuranosyl-3,4,6-tri-*O*-acetyl-α-d-glucopyranoside	*Prunus padus*	[58]
**97**	1,2′,6′-Triacetyl-3,6-diferuloylsucrose	*Polygonum perfoliatum*	[44]
**98**	2′,6′-Diacetyl-3,6-diferuloylsucrose	*Polygonum perfoliatum*; *Smilax china*; *Heterosmilax erythrantha*	[14,44,45]
**99**	1,3-Di-*p*-coumaroyl-6-feruloyl-2′,6′-diacetylsucrose or smilaside F	*Smilax china*	[14]
**100**	Smiglaside B	*Smilax glabra*	[40]
**101**	Smiglaside E	*Smilax china*; *Smilax glabra*	[14,40]
**102**	Vanicoside A	*Polygonum perfoliatum*; *Polygonum pensylvanirum*	[44,53]
**103**	2′-Acetyl-1,6′-diferuloyl-3,6-di-*p*-coumaroyl sucrose or hydropiperoside B	*Polygonum hydropiper* L.	[52]
**104**	2′-*O*-Acetylhydropiperoside or vanicoside C	*Polygonum pensylvanirum*	[49]
**105**	1-*O*-*p*-Coumaroyl-3,6-*O*-diferuloyl-β-d-fructo-furanosyl-(2→1)-(2-*O*-acetyl)-α-d-glucopyranoside or smilaside K	*Smilax bracteata*	[15]
**106**	(1,3-*O*-Di-*p*-coumaroyl-6-*O*-feruloyl)-β-d-fructo-furanosyl-(2→1)-(2-*O*-acetyl)-α-d-glucopyranoside or smilaside H	*Smilax bracteata*	[15]
**107**	3,6-Diferuloyl-2′-acetyl sucrose or smilaside B	*Smilax china*	[14]
**108**	2′,4′,6′-Triacetyl-3,6-diferuloylsucrose or smiglaside C	*Smilax glabra*; *Polygonum perfoliatum*	[40,44]
**109**	β-d-(1-*O*-Acetyl-3,6-*O*-trans-dicinnamoyl)fructo-furanosyl-α-d-(2,4,6-*O*-triacetyl)glucopyranoside or niruriside	*Phyllanthus niruri* L.	[60]
**110**	1,2′,4′,6′-Tetraacetyl-3,6-diferuloylsucrose	*Polygonum perfoliatum*	[44]
**111**	Smiglaside A	*Smilax glabra*	[40]
**112**	Smiglaside D	*Smilax glabra*	[40]
**113**	4′-*O*-Acetylvanicoside A or vanicoside E	*Polygonum pensylvanicum*	[49]
**114**	(3,6-*O*-Diferuloyl)-β-d-fructofuranosyl-(2→1)-(4-*O*-*p*-coumaroyl-2-*O*-acetyl)-α-d-glucopyranoside or quiquesetinerviuside E	*Calamus quiquesetinervius* Burret	[4]
**115**	(3,6-*O*-Diferuloyl)-β-d-fructofuranosyl-(2→1)-(4-*O*-feruloyl-2-*O*-acetyl)-α-d-glucopyranoside or quiquesetinerviuside C	*Calamus quiquesetinervius* Burret	[4]
**116**	3-*O*-*p*-Coumaroyl-β-d-fructofuranosyl2,3,4,6-tetra-*O*-acetyl-α-d-glucopyranoside	*Prunus padus*	[58]
**117**	1-*O*-Acetyl-3-*O*-*p*-coumaroyl-β-d-fructofuranosyl 2,3,6-tri-*O*-acetyl-α-d-glucopyranoside	*Prunus padus*	[58]
**118**	β-d-(1-O-Acetyl-3,6-*O*-*p*-*E*-dicoumaroyl)-fructo-furanosyl-α-d-(4′-*O*-acetyl-2′-*O*-*p*-*E*-coumaroyl)-glucopyranoside	*Froelichia floridana*	[35]
**119**	2-Feruloyl-*O*-α-d-glucopyranoyl-(1′→2)-3,6-*O*-feruloyl-β-d-fructofuranoside	*Paris polyphylla* var. *yunnanensis*	[61]
**120**	3-*O*-Caffeoyl-β-d-fructofuranosyl 2,3,4,6-tetra-*O*-acetyl-α-d-glucopyranoside	*Prunus ssiori*	[24]
**121**	Magnoloside A	*Magnolia obovata* Thunb	[62]
**122**	β-(*p*-Hydroxyphenyl)ethyl *O*-α-l-rhamno-pyranosyl-(1→3)-6-*O*-*trans*-*p*-coumaroyl-β-d-gluco-pyranoside or osmanthuside B_6_	*Osmanthus asiaticus*; *Ligustrum purpurascens*	[22,24]
**123**	β-(*p*-Hydroxyphenyl)ethyl *O*-α-l-rhamno-pyranosyl-(1→3)-4-*O*-*cis*-*p*-coumaroyl-β-d-gluco-pyranoside or osmanthuside D	*Osmanthus asiaticus*	[22]
**124**	Jionoside D	*Rehmannia glutinosa* var. *Purpurea*; *Scrophularia nodosa* L.	[19,63]
**125**	2-Phenylethyl *O*-α-l-rhamnopyranosyl-(1→3)-4-*O*-caffeoyl-β-d-glucopyranoside or jionoside C	*Rehmannia glutinosa* var. *Purpurea*	[19]
**126**	Osmanthuside B	*Ligustrum purpurascens*; *cistanche salsa*	[24,64]
**127**	Lipedoside A-II	*Ligustrum purpurascens*	[24]
**128**	Isoverbascoside	*Lantana camaro* L.; *Pedicularis artselaeri*; *Pedicularis striata*; *Markhamia stipulate*; *Fernandoa adenophylla*; *Markhamia lutea*; *Scrophularia scorodonia*	[15,29,65,66,67,68,69]
**129**		*Scrophularia nodosa* L.	[63]
**130**	6′-*O*-(*E*)-Cinnamoyl verbascoside	*Osmanthus austrocaledonica*	[65]
**131**	Acteoside or verbascoside	*Rehmannia glutinosa* var. *Purpurea*; *Ligustrum purpurascens*; *Calceolaria hypericina*; *Lantana camaro* L.; *Scrophularia nodosa* L.; *Pedicularis artselaeri*; *Pedicularis striata*; *Markhamia stipulate*; *Fernandoa adenophylla*; *Markhamia lutea*; *Scrophularia scorodonia*; *Penstemon serrulatus* Menz; *Aeginetia indica* Linn; *Pedicularis lasiophrys*; *Lagotis stolonifera*; *Conandron ramoidioides*; *Paulownia tomentosa* stem; *Phlomis grandiflora*; *Pedicularis spicata*; *Pedicularis bngijora*; *cistanche salsa*; *Brandisia hancei*; *Phlomis linearis*	[15,19,24,25,29,63,66,67,68,69,70,71,72,73,74,75,76,77,78,79,80,81,82,83,84]
**132**	*cis*-Acteoside or cisacteoside	*Scrophularia ningpoensis* Hemsl; *Scrophularia nodosa* L.; *Penstemon serrulatus Menz*	[17,63,71]
**133**	Cistanoside C or leucosceptoside A or *trans*-leucosceptoside A	*Rehmannia glutinosa* var. *Purpurea*; *Fernandoa adenophylla*; *Penstemon serrulatus* Menz; *Pedicularis bngijora*; *cistanche salsa*; *Lamiophlomis rotata*	[19,69,71,79,85,86]
**134**	*cis*-Leucosceptoside A	*Penstemon serrulatus* Menz	[71]
**135**	2′′,3′′′-Diacetyl acteoside	*Aeginetia indica* Linn	[72]
**136**	2′-Acetyl acteoside	*Rehmannia glutinosa* var. *Purpurea*; *cistanche salsa*; *Aeginetia indica* Linn; *Brandisia hancei*	[19,64,72,82]
**137**	l′-*O*-β-d-(3-Methoxy-4-hydroxy-β-phenyl)-ethyl-6′-*O*-feruloyl-α-l-(2-acetyl)-rhamnosyl-(1→3′)-4′-acetylglucopyranoside or pedicularioside E	*Pedicularis lasiophrys*	[73]
**138**	Martynoside or *trans*-martynoside	*Rehmannia glutinosa* var. *Purpurea*; *Pedicularis artselaeri*; *Fernandoa adenophylla*; *Penstemon serrulatus* Menz; *Paulownia tomentosa* stem; *Galeopws pubescens*	[19,66,69,71,76,87]
**139**	*cis*-Martynoside	*Penstemon serrulatus* Menz	[71]
**140**	2-(4-Hydroxy-3-methoxyphenyl)ethyl *O*-α-l-rhamnopyranosyl-(1→3)-*O*-(4-*O*-feruloyl)-β-d-glucopyranoside or cistanoside D	*cistanche salsa*; *Pedicularis artselaeri*; *Pedicularis lasiophrys*; *Pedicularis bngijora*	[64,66,73,79]
**141**	2-(3′,4′-Dihydroxyphenyl)-ethanol 1-*O*-β-d-xylosyl-(1→3)-β-d-(4-caffeyl)-glucoside or conandroside	*Conandron ramoidioides*	[74]
**142**	Isonuomioside A	*Paraboea glutinosa*; *Lantana camaro* L.	[27,29]
**143**	Calceolarioside E	*Paraboea glutinosa*; *Lantana camaro* L.	[27,29]
**144**	Plantamajoside	*Lagotis stolonifera*	[75]
**145**	Isocistanoside F	*Ligustrum purpurascens*	[24]
**146**	α-l-Rhamnopyranosyl(1→3)-*O*-(4-*O*-caffeoyl)-d-glucopyranoseor cistanoside F	*cistanche salsa*	[85]
**147**	3-Hydroxy-4-methoxy-β-phenylethoxy-*O*-α-l-rhamnopyranosyl-(1→3)-6-*O*-feruloyl-β-d-gluco-pyranoside or isomartynoside	*Galeopws pubescens*	[87]
**148**	1′-*O*-β-d-(3,4-Dihydroxy-β-phenyl)-ethyl-4′-*O*-caffeoyl-β-d-xylopyranosyl-(1′′′→6′)-glucopyran oside or calceolarioside C	*Calceolaria hypericina*	[25]
**149**	4-Cinnamoyl desxylosyl mussatioside	*Mussatia*	[88]
**150**	1-*O*-*trans*-Caffeoyl-2′-*O*-*trans*-sinapoylgentiobiose.	*Wasabia japonica* Matsumura	[89]
**151**	1-*O*-*trans*-Feruloyl-2′-*O*-*trans*-sinapoylgentiobiose	*Wasabia japonica* Matsumura	[89]
**152**	1,2′-di-*O*-*trans*-sinapoylgentiobiose	*Wasabia japonica* Matsumura	[89]
**153**	1-(3′′,4′′-Dihydroxy-5′′-methoxy)-*O*-*trans*-cinnamoyl-2′-*O*-*trans*-feruloyl gentiobiose	*Wasabia japonica* Matsumura	[89]
**154**	1-(3′′,4′′-Dihydroxy-5′′-methoxy)-*O*-*trans*-cinnamoyl-2′-*O*-*trans*-sinapoylgentiobiose	*Wasabia japonica* Matsumura	[89]
**155**	1,2′-Di-(3′′,4′′-dihydroxy-5′′-methoxy)-*O*-*trans*-cinnamoyl gentiobiose	*Wasabia japonica* Matsumura	[89]
**156**	(5-*O*-*E*-Caffeoyl)-β-d-apio-d-furanosyl-(1→6)-β-d-glucopyranosyl benzoic acid ester or psydroside	*Psydrax livida*	[90]
**157**	Crenatoside	*Orobanche crenata*	[91]
**158**	Campneoside II or orobanchoside	*Paulownia tomentosa* stem; *Orobanche crenata*	[76,91]
**159**	Campneoside I	*Paulownia tomentosa* stem	[76]
**160**	Ligurobustoside C	*Ligustrum purpurascens*	[24]
**161**	Ligurobustoside I	*Ligustrum purpurascens*	[24]
**162**	1-*O*-{6-*O*-[3-*O*-(*E*,*E*)-(β,β′-*bis*-Sinapoyl)-β-d-fructo-furanosyl]}-α-d-glucopyranoside intramolecular ester or glomeratose E	*Polygala glomerata*	[38]
**163**	3-*O*-[(*E*)-Sinapoyl]-β-d-fructofuranosyl-(2→1)-[β-d-glucopyranosyl-(1→2)]-[6-*O*-(*E*)-sinapoyl]-α-d-glucopyranosideor tricornose D	*Polygala tricornis*	[37]
**164**	3-*O*-[(*E*)-3,4,5-Trimethoxycinnamoyl]-β-d-fructo-furanosyl-(2→1)-[β-d-glucopyranosyl-(1→2)]-[6-*O*-(*E*)-sinapoyl]-α-d-glucopyranoside or tricornose C	*Polygala tricornis*	[37]
**165**	3-*O*-(*E*)-3,4,5-Trimethoxycinnamoyl-[4-*O*-(*E*)-feruloyl]-β-d-fructofuranosyl-(2→1)-[β-d-gluco-pyranosyl-(1→2)]-[6-*O*-(*E*)-sinapoyl]-α-d- gluco-pyranoside or tricornose F	*Polygala tricornis*	[37]
**166**	3-*O*-(*E*)-3,4,5-Trimethoxycinnamoyl-[4-*O*-(*E*)-sinapoyl]-β-d-fructofuranosyl-(2→1)-[β-d-glucopyranosyl-(1→2)]-[6-*O*-(*E*)-sinapoyl]-α-d-glucopyranoside or tricornose E	*Polygala tricornis*	[37]
**167**	Reiniose E	*Polygala reinii* Fr.*et* Sav	[46]
**168**	Reiniose F	*Polygala reinii* Fr.*et* Sav	[46]
**169**	*O*-β-d-Glucopyranosyl-(1→3)-6-*O*-feruloyl -α-d-glucopyranosyl β-d-fructofuranoside or arillatose C	*Polygala arillata*	[51]
**170**	*O*-β-d-Glucopyranosyl-(1→3)-6-*O*-sinapoyl-α-d-glucopyranosyl β-d-fructofuranoside or arillatose D	*Polygala arillata*	[51]
**171**	*O*-β-d-Glucopyranosyl-(1→3)-α-d-gluco-pyranosyl-3′-O-feruloyl-β-d-fructofuranoside or arillatose E	*Polygala arillata*	[51]
**172**	*O*-β-d-Glucopyranosyl-(1→3)-α-d-gluco-pyr anosyl-3′-O-sinapoyl-β-d-fructofuranoside or arillatose F	*Polygala arillata*	[51]
**173**	3-Feruloyl-4-acetyl-6′-(13′-*O*-β-d-gluco-pyranosyl)feruloylsucrose	*Lilium longiflorum*	[55]
**174**	Reiniose D	*Polygala reinii* Fr.*et* Sav; *Polyyala fallax*	[46,92]
**175**	Dalmaisiose A	*Polygala dalmaisiana*	[93]
**176**	3,4-Dihydroxyphenylethanol-6-*O*-*trans*-caffeoyl-β-d-apiofuranosyl(1→5)-β-d-apiofuranosyl(1→3)-β-d-glucopyranoside or paraboside B	*Paraboea glutinosa*	[27]
**177**	3,4-Dihydroxyphenylethanol-4-*O*-*trans*-caffeoyl-β-d-apiofuranosyl(1→5)-β-d-apiofuranosyl(1→3)-β-d-glucopyranosideor paraboside A	*Paraboea glutinosa*	[27]
**178**	2-(3,4-Dihydroxyphenyl)ethyl 3,6-*O*-*bis*(β-d-apiofranosyl)-4-*O*-caffeoyl-β-d-glucopyranoside or paucifloside	*Lysionotus pauciflorus*	[94]
**179**	l′-*O*-β-d-(3,4-Dihydroxy-β-phenyl)-ethyl-4′-*O*-caffeoyl-β-d-apiosyl-(l→3′)-α-l-rhamnosyl-(l→6′)-glucopyranoside or pedicularioside A	*Pedicularis striata*; *Markhamia lutea*; *Pedicularis striata pall* ssp. *arachnoidea*; *Pedicularis spicata*	[5,67,77,78]
**180**	l′-*O*-β-d-(3,4-Dihydroxy-β-phenyl)-ethyl-4′-*O*-feruloyl-β-d-apiosyl(1→3′)-α-l-rhamnosyl-(1→6′)-glucopyranoside or pedicularioside M	*Pedicularis striata pall* ssp. *arachnoidea*	[77]
**181**	l′-*O*-β-d-(3-hydroxy-4-methoxy-β-phenyl)-ethyl-4′-feruloyl-β-d-apiosyl(l→3′)-α-l-rhamnosyl-(l→6′)-glucopyranoside or pedicularioside N	*Pedicularis artselaeri*; *Pedicularis striata pall* ssp. *arachnoidea*	[66,77]
**182**	l′-*O*-β-d-(3-Methoxy-4-hydroxy-β-phenyl)-ethyl-4′-*O*-feruloyl-β-d-apiosyl-(1→3′)-α-l-rhamnos yl-(1→6′)-glucopyranoside or pedicularioside H	*Pedicularis spicata*	[78]
**183**	3,4-Dihydroxy-β-phenylethoxy-*O*-[α-arabino-pyranosyl-(1′′′′→2′′)-α-rhamnopyranosyl-(1′′′→3′′)-6′′-*O*-caffeoyl-β-glucopyranoside] or markhamioside C	*Markhamia stipulata*	[68]
**184**	Ehrenoside	*Veronica pectinata* var. *glandulosa; Aragoa cundinamarcensis*	[75,95,96]
**185**	3,4-Dihydroxy-β-phenylethoxy-*O*-[α-arabino-pyranosyl-(1′′′′→2′′)-α-rhamnopyranosyl-(1′′′→3′′)-4-*O*-caffeoyl-6-*O*-acetyl-β-glucopyranoside or markhamioside D	*Markhamia stipulata*	[68]
**186**	2-(3,4-Dihydroxyphenyl)ethyl-*O*-α-l-arabino-pyranosyl-(1→2)-[α-l-rhamnopyranosyl-(1→3)]-(4-*O*-*trans*-feruloyl)-β-d-glucopyranoside or verpectoside A	*Veronica pectinata* var. *glandulosa*	[95]
**187**	Lagotoside	*Lagotis stolonifera*	[75]
**188**	3,4-Dihydroxy-β-phenylethoxy-*O*-β-apiofuranosyl-(1→2)-α-rhamnopyranosyl-(1→3)-4-*O*-caffeoyl-β-glucopyranosideor 2′′-*O*-β-apiosylverbascoside	*Markhamia stipulata* ; *Fernandoa adenophylla*	[68,69]
**189**	1-*O*-(3,4-Dihydroxyphenyl)ethyl β-d-apiofuranosyl(1→2)-α-l-rhamnopyranosyl (1→3)-4-*O*-caffeoyl-6-acetyl-β-d-glucopyrano sideor luteoside A	*Markhamia stipulate*; *Markhamia lutea*	[5,68]
**190**	1-*O*-(3,4-Dihydroxyphenyl)ethyl β-d-apio-furanosyl(1→2)-α-l-rhamnopyranosyl(1→3)-6-*O*-caffeoyl-β-d-glucopyranosideor luteoside B	*Markhamia stipulate*; *Markhamia lutea*	[5,68]
**191**	1-*O*-(3,4-Dihydroxyphenyl)ethyl β-d-apio-furan osyl(1→2)-α-l-rhamnopyranosyl(1→3)-6-*O*-feruloyl-β-d-glucopyranoside or luteoside C	*Markhamia lutea*	[5]
**192**	3-Hydroxy-4-methoxy-β-phenylethoxy-*O*-[β-apio-furanosyl-(1′′′′→2′′)-α-rhamnopyranosyl-(1′′′→3′′)-6′′-*O*-feruloyl-β-glucopyranoside] or markhamioside B	*Markhamia stipulate*	[68]
**193**	3,4-Dihydroxy-β-phenylethoxy-*O*-[β-galacto-pyranosyl-(1′′′′→2′′)-α-rhamnopyranosyl-(1′′′→3′′)-4-*O*-caffeoyl-6-*O*-acetyl-β-glucopyranoside] or markhamioside E	*Markhamia stipulate*	[68]
**194**	2-(3,4-Dihydroxyphenyl)ethyl-*O*-β-d-gluco-pyranosyl-(1→2)-[α-l-rhamnopyranosyl-(1→3)]-(4-*O*-*trans*-caffeoyl)-β-d-glucopyranoside or verpectoside B	*Veronica pectinata* var. *glandulosa*	[95]
**195**	2-(3,4-Dihydroxyphenyl)ethyl-*O*-β-d-gluco-pyranosyl-(1→2)-[α-l-rhamnopyranosyl-(1→3)]-(4-*O*-*trans*-feruloyl)-β-d-glucopyranoside or verpectoside C	*Veronica pectinata* var. *glandulosa*	[95]
**196**	1′-*O*-β-d-(3-Methoxy-4-hydroxy-phenyl)-ethyl-α-l-rhamnosyl-(1→3′)-α-l-arabinosyl-(1→4′)-6′-*O*-feruloyl-glucopyranoside or pedicularioside I	*Pedicularis bngijora*	[79]
**197**	Angoroside A	*Scrophularia nodosa L.; Scrophularia scorodonia*	[63,70]
**198**	Scrophuloside B_1_	*Scrophularia nodosa* L.	[63]
**199**	Scrophuloside B_2_	*Scrophularia nodosa* L.	[63]
**200**	3,4-Dihydroxy-β-phenylethoxy-*O*-α-l-arabino-pyranosyl-(1→6)-α-l-rhamnopyranosyl-(1→3)-4-*O*-feruloyl-β-d-glucopyranoside or angoroside D	*Scrophularia scorodonia*	[70]
**201**	Angoroside C	*Scrophularia nodosa* L.	[63]
**202**	Forthysioside B	*Markhamia lutea*	[5]
**203**	6′-β-d-Apiofuranosyl cistanoside C	*Lamiophlomis rotata*	[86]
**204**	Lamiophlomiside A	*Lamiophlomis rotata*	[86]
**205**	*cis*-Lamiophlomiside A	*Lamiophlomis rotata*	[86]
**206**	Forsythoside B	*Phlomis grandiflora*; *Phlomis fruticosa*	[80]
**207**	Alyssonoside	*Phlomis grandiflora*; *Phlomis fruticosa*	[80]
**208**	2-(3,4-Dihydroxyphenyl)ethyl *O*-α-rhamno-pyranosyl-(1→3)-[β-d-galactopyranosyl-(l→6)]-(4-*O*-*p*-coumaroyl)-β-d-glucopyranoside or jionoside E	*Rehmannia glutinosa* var. *Purpurea*	[19]
**209**	Purpureaside C	*Rehmannia glutinosa* var. *Purpurea*; *Scrophularia nodosa* L.	[19,63]
**210**	Jionoside A_1_	*Rehmannia glutinosa* var. *Purpurea*	[19]
**211**	Jionoside A_2_	*Rehmannia glutinosa* var. *Purpurea*	[19]
**212**	Jionoside B_1_	*Rehmannia glutinosa* var. *Purpurea*	[19]
**213**	Jionoside B_2_	*Rehmannia glutinosa* var. *Purpurea*	[19]
**214**	Echinacoside	*Ligustrum purpurascens*; *cistanche salsa*	[24,81]
**215**	2-(4-Hydroxy-3-methoxyphenyl)ethyl *O*-α-l-rhamnopyranosyl-(1→3)-*O*-[β-d-glucopyrano syl(1→6)]-(4-*O*-caffeoyl)-β-d-glucopyranosideor cistanoside A	*Ligustrum purpurascens*	[81]
**216**	2-(4-Hydroxy-3-methoxyphenyl)ethyl *O*-α-l-rhamnopyranosyl-(1→3)-*O*-[β-d-glucopyran osyl(1→6)]-(4-*O*-feruloyl)-β-d-glucopyranoside or cistanoside B	*Ligustrum purpurascens*	[81]
**217**	Poliumoside	*Brandisia hancei*	[82]
**218**	[β-(3′,4′-Dihydroxylphenyl)-ethyl]-(2-*O*-acetyl)-(3,6-*O*-di-α-l-rhamnopyranosyl-(4-*O*-caffeoyl)β-d-glucopyranoside or brandioside	*Brandisia hancei*	[82]
**219**	Arenarioside	*Scrophularia nodosa* L.	[63]
**220**	1-*O*-3,4-(Sihydroxyphenyl)-ethyl-β-d-apiofuranosyl-(1→4)-α-l-rharmnopyranosyl-(1→3)-4-*O*-caffeoyl-β-d-glucopyranoside or myricoside	*Markhamia lutea*; *Picria tel*-*ferae* Lour.	[5,97]
**221**	Rossicaside B	*Boschniakia rossica*	[98]
**222**	Rossicaside A	*Boschniakia rossica*	[98]
**223**	2-*O*-Acetylrossicaside A	*Ortbocarpus densiflourus* var. *gracilis*	[99]
**224**	β-d-glucopyranosyl(1→4)-α-l-rhamnopyranosyl-(1→3)-(4-*O*-*trans*-caffeoyl)-d-glucopyranose	*Boschniakia rossica*	[98]
**225**	Lavandulifolioside	*leonurus glaucescens*	[83]
**226**	β-(3,4-Dihydroxyphenyl)-ethyl-O-α-L-arabinopyranosyl-(1→2)-α-L-rhamnopyranosyl-(l →3)-4-O-feruloyl-β-D-glucopyranoside or leonosides A	*leonurus glaucescens*	[83]
**227**	β-(3-Hydroxy,4-methoxyphenyl)-ethyl-*O*-α-l-arabinopyranosyl-(l→2)-α-l-rhamnopyranosyl-(1→3)-4-*O*-feruloyl-β-d-glucopyranoside or leonoside B	*leonurus glaucescens*	[83]
**228**	2R-Galactosyl-acteoside or lamalboside	*Lamium album*	[100]
**229**	3,4-Dihydroxy-β-phenylethoxy-*O*-β-d-gluco-pyranosyl-(1→2)-α-l-rhamnopyranosyl-(1→3)-4-*O*-caffeoyl-β-d-glucopyranoside or phlinoside A	*Phlomis linearis*	[84]
**230**	3,4-Dihydroxy-β-phenylethoxy-*O*-α-l-lyxo-pyranosyl-(1→2)-α-l-rhamnopyranosyl-(1→3)-4-*O*-caffeoyl-β-d-glucopyranoside or teucrioside	*Teucrium chamaedrys*	[101]
**231**	3,4-Dihydroxy-β-phenylethoxy-*O*-β-d-xylo-pyranosyl-(1→2)-α-L-rhamnopyranosyl-(1→3)-4-*O*-caffeoyl-β-d-glucopyranoside or phlinoside B	*Phlomis linearis*	[84]
**232**	3,4-Dihydroxy-β-phenylethoxy-*O*-β-d-xylo-pyranosyl-(1→2)-α-l-rhamnopyranosyl-(1→3)-4-*O*-feruloyl-β-d-glucopyranoside or phlinoside D	*Phlomis lineuris*	[102]
**233**	2-(4-Hydroxyphenyl)-ethyl-[3-*O*-α-l-rhamno-pyranosyl(1→4)-α-l-rhamnopyranosyl][6-*O*-*p*-coumaroyl]-*O*-β-d-glucopyranoside or ligupurpuroside C	*Ligustrum purpurascens*	[24]
**234**	2-(4-Hydroxyphenyl)-ethyl-[3-*O*-α-l-rhamno-pyranosyl(1→4)-α-l-rhamnopyranosyl][6-*O*-(*E*)-caffeoyl]-*O*-β-d-glucopyranoside or ligupurpuroside D	*Ligustrum purpurascens*	[24]
**235**	3-*O*-[α-l-Rhamnopyranosyl(1→4)-α-l-rhamno-pyranosyl]-4-*O*-(*E*)-caffeoyl-d-glucopyranose or ligupurpuroside F	*Ligustrum purpurascens*	[24]
**236**	Ligupurpuroside B	*Ligustrum purpurascens*	[24]
**237**	Ligurobustosides N	*Ligustrum purpurascens*	[24]
**238**	Ligupurpuroside A	*Ligustrum purpurascens*	[24]
**239**	3,4-Dihydroxy-β-phenylethoxy-*O*-α-l-rhamno-pyranosyl-(l→2)-α-l-rhamnopyranosyl-(1→3)-4-*O*-caffeoyl-β-d-glucopyranoside or phlinoside C	*Phlomis linearis*	[84]
**240**	3,4-Dihydroxy-β-phenylethoxy-*O*-α-l-rhamno-pyranosyl-(l→2)-α-l-rhamnopyranosyl-(l→3)-4-*O*-feruloyl-β-d-glucopyranoside or phlinoside E	*Phlomis lineuris*	[102]
**241**	Myricoside	*Clerodendrum serratum*	[103]
**242**	3-Hydroxy-4-methoxy-β-phenethyl-*O*-β-d-apio-furanosyl-(1→3)-α-l-rhamnopyranosyl-(1→3)-4-*O*-feruloyl-β-d-glucopyranoside or serratumoside A	*Clerodendrum serratum*	[103]
**243**	Aragoside	*Aragoa cundinamarcensis*	[96]
**244**	Persicoside	*Aragoa cundinamarcensis*	[96]
**245**	1′-*O*-β-d-(3-Hydroxy-4-methoxy-β-phenyl)-ethyl-4′-*O*-feruloyl-β-d-glucopyranosyl-(1→3)-α-l-rhamnosyl-(1→6′)-glucopyranoside or artselaeroside B	*Pedicularis artselaeri*	[66]
**246**	3,4-Dihydroxy-β-phenyl-ethyl-*O*-α-l-rhamno-pyranosyl-(1→2)-*O*-β-d-glucopyranosyl-(1→6)-3-*O*-caffeoyl-β-d-allopyranoside or magnoloside B	*Magnolia obovata* Thunb	[62]
**247**	α-l-Xylopyranosyl-(4′′→2′)-(3-*O*-β-d-gluco-pyranosyl)-1′-*O*-*E*-caffeoyl-β-d-glucopyranoside	*Coussarea hydrangeifolia*	[21]
**248**	2-(3,4-Dihydroxyphenyl)-*R*,*S*-2-ethoxyethyl-*O*-β-d-glucopyranosyl(1→4)-α-l-rhamno-pyranosyl(1→3)(4-*O*-*trans*-caffeoyl)-β-d-gluco-pyranoside or rossicaside F	*Boschniakia rossica*	[97]
**249**	4-Cinnamoyl desxylosylmussatioside	*Mussatia*	[88]
**250**	4-*p*-Coumaroylmussatioside	*Mussatia*	[88]
**251**	4-*cis*-*p*-Coumaroylmussatioside	*Mursatia byacinthima*	[104]
**252**	4-*p*-Methoxycmnamoylmussatioslde ormussatloside III	*Mussatia*	[88]
**253**	4-Feruloylmussatioside	*Mussatia*	[88]
**254**	4-Dimethylcaffeoylmussatloside or mussatioside II	*Mussatia*	[88]
**255**	3-*O*-[(*E*)-Sinapoyl]-β-d-fructofuranosyl-(2→1)-[β-d-glucopyranosyl-(1→4)-β-d-gluco-pyranosyl-(1→2)]-[6-*O*-(*E*)-sinapoyl]-α-d-gluco-pyranoside or tricornose G	*Polygala tricornis*	[37]
**256**	3-*O*-(*E*)-Sinapoyl-[4-*O*-(*E*)-*p*-coumaroyl]-β-d-fructofuranosyl-(2→1)-[β-d-glucopyranosyl-(1→4)-β-d-glucopyranosyl-(1→2)]-[6-*O*-(*E*)-sinapoyl]-α-d-glucopyranoside or tricornose L	*Polygala tricornis*	[37]
**257**	3-*O*-(*E*)-Sinapoyl-[4-*O*-(E)-feruloyl]-β-d-fructo-furanosyl-(2→1)-[β-d-glucopyranosyl-(1→4)-β-d-glucopyranosyl-(1→2)]-[6-*O*-(*E*)-sinapoyl] -α-d-glucopyranoside or tricornose K	*Polygala tricornis*	[37]
**258**	3-*O*-(*E*)-sinapoyl-[4-*O*-(*E*)-sinapoyl]-β-d-fructo-furanosyl-(2→1)-[β-d-glucopyranosyl-(1→4)-β-d-glucopyranosyl-(1→2)]-[6-*O*-(*E*)-sinapoyl]-α-d-glucopyranoside or tricornose H	*Polygala tricornis*	[37]
**259**	3-*O*-(*E*)-3,4,5-Trimethoxylcinnamoyl-[4-*O*-(*E*)-feruloyl]-β-d-fructofuranosyl-(2→1)-[β-d-gluco-pyranosyl-(1→4)-β-d-glucopyranosyl-(1→2)]-[6-*O*-(*E*)-sinapoyl]-α-d-glucopyranoside or tricornose J	*Polygala tricornis*	[37]
**260**	3-*O*-(*E*)-3,4,5-Trimethoxylcinnamoyl-[4-*O*-(*E*)-sinapoyl]-β-d-fructofuranosyl-(2→1)-[β-d-glucopyranosyl-(1→4)-β-d-glucopyranosyl-(1→2)]-[6-*O*-(*E*)-sinapoyl]-α-d-glucopyranoside or tricornose I	*Polygala tricornis*	[37]
**261**	Senegose I	*Polygala senega* var. *latifolia* Torr. Et Gray	[105]
**262**	1-*O*-(*E*)-*p*-Coumaroyl-(3-*O*-benzoyl)-β-d-fructo-furanosyl-(2→1)-[β-d-glucopyranosyl-(1→2)]-[6-*O*-acetyl-β-d-glucopyranoysl-(1→3)]-[4-*O*-(*E*)-feruloyl]-(6-d-acetyl)-α-d-glucopyranoside or glomeratose F	*Polygala glomerata*	[38]
**263**	1-*O*-(*E*)-*p*-Coumaroyl-(3-*O*-benzoyl)-β-d-fructo-furanosyl-(2→l)-[β-d-glucopyranosyl-(1→2)]-[6-*O*-acetyl-β-d-glucopyranoside-(1→3)]-{4-*O*-[4-*O*-β-d-glucopyranosyl-(*E*)-feruloyl]}-[6-*O*-(*E*)-*p*-coumaroyl]-α-d-glucopyranosyl or glomeratose G	*Polygala glomerata*	[38]
**264**	1-*O*-*p*-coumaroyl-(3-*O*-benzoyl)-β-d-fructo-furanosyl-(2→1)-[β-d-glucopyranosyl-(1→2)]-[6-*O*-acetyl-β-d-glucopyranosyl-(1→3)]-(4-*O*-*p*-coumaroyl)-α-d-glucopyranoside or fallaxose C	*Polyyala fallax*	[92]
**265**	Reiniose G	*Polygala glomerata; Polygala reinii* Fr. *et* Sav	[38,46]
**266**	Dalmaisiose H	*Polygala dalmaisiana*	[93]
**267**	1-*O*-*p*-Coumaroyl-(3-*O*-benzoyl)-β-d-fructo-furanosyl-(2→1)-[β-d-glucopyranosyl-(1→2)]-[6-*O*-acetyl-β-d-glucopyranosyl-(1→3)]-(4-*O*-feruloyl)-α-d-glucopyranoside or fallaxose D	*Polyyala fallax*	[92]
**268**	Dalmaisiose J	*Polygala dalmaisiana*	[93]
**269**	Dalmaisiose L	*Polygala dalmaisiana*	[93]
**270**	Dalmaisiose M	*Polygala dalmaisiana*	[93]
**271**	Reiniose H	*Polygala reinii* Fr. *et* Sav	[46]
**272**	Senegose G	*Polyyala fallax*; *Polygala senega* var. *latifolia* Torr. Et Gray	[92,105]
**273**	Senegose H	*Polygala senega* var. *latifolia* Torr. Et Gray	[105]
**274**	Senegose F	*Polygala reinii* Fr. *et* Sav; *Polygala senega* var. *latifolia* Torr. Et Gray	[46,105]
**275**	3-*O*-*β*-D-Glucopyranosylpresenegenin 28-*O*-*β*-D-xylopyranosyl-(1→4)-*α*-L-rhamnopyranosyl-(1→2)-{4-*O*-[(*E*)-3,4-dimethoxycinnamoyl]}-*β*-D-fucopyranosyl ester or Polygalasaponin XLII	*Polygala glomerata Lour*	[106]
**276**	3,4-Dihydroxy-β-phenylethyl-*O*-α-l-rhamno-pyranosyl-(1→2)-*O*-[*O*-β-d-glucopyranosyl-(1→4)-β-d-glucopyranosyl-(1→6)]-3-*O*-caffeoyl-β-d-allopyranoside or magnoloside C	*Magnolia obovata* Thunb	[62]
**278**	3-*O*-{4-*O*-[β-d-Glucopyranosyl-(1→3)-(2-*O*-acetyl)-α-l-rhamnopyranosyl]-feruloyl}-β-d-fructo-furanosyl-(2→1)-(4,6-di-*O*-benzoyl)-α-d-gluco-pyranoside or fallaxose B	*Polyyala fallax*	[92]
**279**	2-(3,4-Dihydroxyphenyl)ethyl *O*-β-apio-furanosyl-(1→6)-*O*-[*O*-β-apiofuranosyl-(1→4)-α-rhamnopyranosyl-(1→3)]-4-*O*-(*E*)-caffeoyl-β-glucopyranoside or lunariifolioside	*Phlomis lunariifolia*	[106]
**280**	Tenuifoliose K	*Polygala tenuifolia* Willd	[11]
**281**	Tenuifoliose J	*Polygala tenuifolia* Willd	[11]
**282**	tenuifoliose I	*Polygala tenuifolia* Willd	[11]
**283**	Tenuifoliose H	*Polygala tenuifolia* Willd	[11]
**284**	Tenuifoliose C	*Polygala tenuifolia* Willd; *Polyyala fallax*	[12,92]
**285**	Tenuifoliose B	*Polygala tenuifolia* Willd	[12]
**286**	Tenuifoliose D	*Polygala tenuifolia* Willd; *Polygala reinii* Fr. *et* Sav	[12,46]
**287**	Tenuifoliose E	*Polygala tenuifolia* Willd	[12]
**288**	Tenuifoliose A	*Polygala tenuifolia* Willd	[11,12]
**289**	Tenuifoliose P	*Polygala tenuifolia* Willd	[11]
**290**	Tenuifoliose O	*Polygala tenuifolia* Willd	[11]
**291**	Reiniose I	*Polygala reinii* Fr. *et* Sav	[46]
**292**	Tenuifoliose N	*Polygala tenuifolia* Willd	[11]
**293**	Reiniose J	*Polygala reinii* Fr. *et* Sav	[46]
**294**	1-*O*-Feruloyl-(3-*O*-benzoyl)-β-d-fructofuranosyl-(2→1)-[β-d-glucopyranosyl-(1→2)]-[β-d-gluco-pyranosyl-(1→3)-(6-*o*-acetyl)-β-d-gluco-pyranosyl-(1→3)]-(6-*o*-feruloyl)-α-d-glucopyranoside or fallaxose E	*Polyyala fallax*	[92]
**295**	Senegose K	*Polygala senega* L.	[107]
**296**	Senegose J	*Polygala senega* L.	[107]
**297**	Senegose N	*Polygala senega* L.	[107]
**298**	Senegose O	*Polygala senega* L.	[107]
**299**	Senegose M	*Polygala senega* L.	[107]
**300**	Senegose L	*Polygala senega* L.	[107]
**301**	Senegose D	*Polygala senega* var. *latifolia* Torr. Et Gray	[108]
**302**	Senegose C	*Polygala senega* var. *latifolia* Torr. Et Gray	[108]
**303**	Senegose B	*Polygala senega* var. *latifolia* Torr. Et Gray	[108]
**304**	Senegose A	*Polygala senega* var. *latifolia* Torr. Et Gray	[108]
**305**	Senegose E	*Polygala senega* var. *latifolia* Torr. Et Gray	[108]
**306**	Dalmaisiose D	*Polygala dalmaisiana*	[93]
**307**	Dalmaisiose B	*Polygala dalmaisiana*	[93]
**308**	Dalmaisiose E	*Polygala dalmaisiana*	[93]
**309**	Dalmaisiose I	*Polygala dalmaisiana*	[93]
**310**	Dalmaisiose N	*Polygala dalmaisiana*	[93]
**311**	Dalmaisiose F	*Polygala dalmaisiana*	[93]
**3312**	Dalmaisiose P	*Polygala dalmaisiana*	[93]
**313**	Dalmaisiose G	*Polygala dalmaisiana*	[93]
**314**	Dalmaisiose C	*Polygala dalmaisiana*	[93]
**315**	Dalmaisiose K	*Polygala dalmaisiana*	[93]
**316**	Dalmaisiose O	*Polygala dalmaisiana*	[93]
**317**	*E*-Senegasaponin b	*Polygala senega* L.var. *latifolia* Torrey *et* Gray	[109]
**318**	*Z*-Senegasaponin b	*Polygala senega* L.var. *latifolia* Torrey *et* Gray	[109]
**319**	Senegin II	*Polygala glomerata* Lour	[106]
**320**	(*Z*)-Senegin II	*Polygala glomerata* Lour	[106]
**321**	Tenuifoliose M	*Polygala tenuifolia* Willd	[11]
**322**	Tenuifoliose L	*Polygala tenuifolia* Willd	[11]
**323**	Tenuifoliose G	*Polygala tenuifolia* Willd	[11]
**324**	Tenuifoliose F	*Polygala tenuifolia* Willd	[11,12]
**325**	3-*O*-β-d-Glucopyranosylpresenegenin 28-*O*-β-d-galactopyranosyl-(1→4)-β-d-xylo-pyranosyl-(1→4)-α-l-rhamnopyranosyl-(1→2)[β-d-glucopyranosyl-(1→3)]-(4-*O*-[(*E*)-3,4-dimethoxycinnamoyl]}-8-*O*-fucopyranosyl ester or polygalasaponin XLIV	*Polygala glomerata* Lour	[106]
**326**	3-*O*-β-d-Glucopyranosylpresenegenin 28-*O*-β-d-galactopyranosyl-(1→4)-β-d-xylopyranosyl-(1→4)-α-l-rhamnopyranosyl-(1→2)-[6-*O*-acetyl-β-d-glucopyranosyl-(1→3)]-{4-*O*-[(*E*)-3,4-dimethoxycinnamoyl])-β-d-fucopyranosyl ester or polygalasaponin XLV	*Polygala glomerata* Lour	[106]
**327**	3-*O*-β-d-Glucopyranosylpresenegenin 28-*O*-β-d-galactopyranosyl-(1→4)-β-*O*-xylopyranosyl-(1→4)-α-l-rhamnopyranosyl-(1→2)-[6-*O*-acetyl-β-d-glucopyranosyl-(1→3)]-{4-*O*-[(*Z*)-3,4-dimethoxycinnamoyl]}-β-d-fucopyranosyl ester or polygalasaponin XLVI	*Polygala glomerata* Lour	[106]
**328**	3-*O*-β-d-Glucopyranosylpresenegenin, 28-*O*-β-d-galactopyransyl(1→4)-β-d-xylopyranosyl -(1→4)-α-l-rhamnopyranosyl-(1→2)-{4-*O*-*p*-methoxycinnamoyl]}-[β-d-glucopyranosy l(1→3)]-β-d-fucopyranosyl ester or polygalasaponin X X X	*Polygala japonica* Houtt.	[56]
**329**	3-*O*-β-d-Glucopyranosylpresenegenin 28-*O*-β-d-galactopyranosyl-(1→4)-β-d-xylo-pyranosyl-(1→4)-α-l-rhamnopyranosyl-(1→2)-[α-l-arabinopyranosyl-(1→3)]-[4-*O*-(*E*)-*p*-methoxycinnamoyl]-β-d-fucopyranosyl ester or polygalasaponin XLIII	*Polygala glomerata* Lour	[106]
**330**	3-*O*-β-d-Glucopyranosylpresenegenin 28-*O*-α-l-arabinopyransyl (1→4)-β-d-xylopyranosyl-(1→4)-[β-d-apiofuranosyl-(1→3)]-α-l-rhamnopyranosyl-(1→2)-[4-*O*-3,4,5-trimethoxy-cinnamoyl]-β-d-fucopyranosyl ester or polygalasaponin XXXI	*Polygala japonica* Houtt.	[56]
**331**	*E*-Senegasaponin a	*Polygala senega* L.var. *latifolia* Torrey *et* Gray	[109]
**332**	*Z*-Senegasaponin a	*Polygala senega* L.var. *latifolia* Torrey *et* Gray	[109]
**333**	1-*O*-(*E*)-*p*-Coumaroyl-(3-*O*-benzoyl)-β-d-fructo-furanosyl-(2→1)-[6-*O*-(*E*)-feruloyl-β-d-gluco-pyranosyl-(1→2)]-[6-*O*-acetyl-β-d-gluco-pyranosyl-(1→3)-(4-*O*-acetyl)-β-d-glucopyranosyl-(1→3)]-4-*O*-[4-*O*-α-l-rhamnopyranosyl-(*E*)-*p*-coumaroyl]-α-d-glucopyranoside or polygalajaponicose I	*Polygala japonica*	[110]
**334**	3-*O*-β-d-Glucopyranosylpresenegenin 28-*O*-α-L-arabinopyransyl-(1→4)-β-d-xylo-pyranosyl-(1→4)-[β-d-apiofuranosyl-(1→3)]-α-l-rhamnopyranosyl-(1→2)-[4-*O*-*p*-methoxy-cinnamoyl]-[α-l-rhamnopyranosyl(1→3)]-β-d-fucopyranosyl ester or polygalasaponin XXXII	*Polygala japonica* Houtt.	[56]
